# Polarization Holographic 4D Phenotyping Reveals Nanoplastic Induced Dysfunction in Zooplankton

**DOI:** 10.1002/advs.77832

**Published:** 2026-10-04

**Authors:** Yuxing Li, Yanmin Zhu, Jingyan Chen, Rongzhou Chen, Yuen‐Wa Ho, Chutian Wang, James Kar‐Hei Fang, Kevin K. Tsia, Edmund Y. Lam

**Affiliations:** ^1^ Department of Electrical and Computer Engineering The University of Hong Kong Pokfulam Hong Kong China; ^2^ Department of Food Science and Nutrition The Hong Kong Polytechnic University Hung Hom Hong Kong China; ^3^ State Key Laboratory of Marine Environmental Health City University of Hong Kong Kowloon Tong Hong Kong China; ^4^ PolyU‐BGI Joint Research Centre for Genomics and Synthetic Biology in Global Ocean Resources The Hong Kong Polytechnic University Hung Hom Hong Kong China; ^5^ Advanced Biomedical Instrumentation Centre Hong Kong Science Park New Territories Hong Kong China

**Keywords:** computational reconstruction, label‐free imaging, multiscale phenotyping, nanoplastic contaminant, polarization holography

## Abstract

Assessing contaminant‐induced dysfunction in complex living systems is critical, but remains technically challenging. Current methods rely predominantly on destructive endpoint readouts or low‐dimensional measurements, which often fail to detect early sublethal changes in freely behaving organisms. Here, we develop Polarization Holographic Imaging Microscopy (PHIM), a multiscale phenotyping framework that integrates polarization‐sensitive holography with computational volumetric reconstruction for label‐free analysis of living zooplankton in natural seawater. Without mechanical scanning, PHIM resolves four‐dimensional (4D) locomotor dynamics and endogenous structural anisotropy. Using marine rotifers as a model zooplankton organism, we show that exposure to polystyrene nanoplastics spanning 20–500 nm does not simply suppress motility, but redistributes organisms into distinct locomotor regimes characterized by altered exploration, reorientation dynamics, and behavioral phenotypes. In parallel, the polarization contrast associated with the digestive vesicles was markedly reduced and spatially associated with fluorescence‐validated nanoplastic accumulation. Orthogonal calcium imaging further reveals accompanying physiological perturbations. These findings establish polarization holographic 4D phenotyping as a scalable and sensitive platform for multiscale assessment of contaminant‐induced dysfunction in living aquatic organisms.

## Graphical abstract

Contaminant‐induced dysfunction in complex aquatic organisms remains difficult to resolve across scales. Polarization Holographic Imaging Microscopy (PHIM) integrates polarization‐sensitive holography with computational volumetric reconstruction for label‐free phenotyping of zooplankton in natural seawater, revealing that nanoplastic stress drives coordinated functional reorganization across 4D locomotion behavior, endogenous structural anisotropy, and physiological state rather than simply suppressing motility.

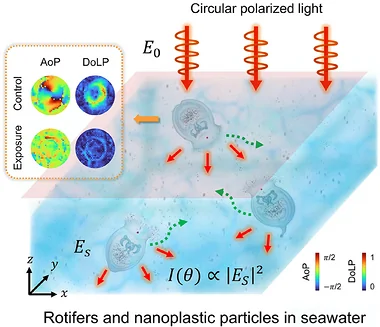

## 1 Introduction

Nanoplastics have emerged as a pervasive stressor in aquatic environments, yet their biologically relevant impacts remain challenging to resolve at the early stages of dysfunction [1, 2]. Current environmental risk assessment relies predominantly on endpoint assays [3, 4], destructive biochemical markers [5, 6], or low‐dimensional measurements [7, 8], all of which compress the dynamics of living systems into static readouts obtained only after substantial dysfunction has already developed. Such strategies are poorly suited to detect early sublethal changes, even though these early perturbations may represent the first manifestations of contaminant stress in real ecosystems [9]. For complex aquatic organisms in particular, contaminant exposure may not initially appear as overt lethality or simple activity suppression, but rather as a progressive reorganization of organismal function across movement, internal structural integrity, and physiological regulation [10, 11]. Resolving this transition from apparently intact behavior to incipient dysfunction remains a central challenge for environmentally relevant phenotyping of living systems.

A major reason this challenge persists is that current measurement approaches do not adequately capture the multiscale nature of contaminant‐induced dysfunction in freely behaving organisms. Conventional behavioral assays are commonly based on two‐dimensional (2D) imaging [12, 13, 14], and therefore overlook the three‐dimensional (3D) structure, temporal state transitions, and heterogeneity of locomotor behavior. At the same time, biochemical and omics‐based measurements provide important molecular information but are typically destructive, population‐averaged, or disconnected from the intact dynamics of living individuals. Spectroscopic tools such as Raman [15, 16, 17, 18] and Fourier‐transform infrared (FTIR) spectroscopy [19, 20] offer chemical specificity for plastic particles [21], while fluorescence imaging can visualize labeled materials or downstream responses [22], yet these methods generally lack the combination of label‐free contrast, volumetric continuity, and compatibility with freely moving organisms needed to interrogate how contaminant burden relates to organismal function in situ [23, 24]. Recent advances in digital holographic and interferometric imaging methods have enabled volumetric observation of small organisms [25] and individual cells [26], but these approaches mainly capture motion, morphology, and phase‐related contrast [27], with limited access to endogenous structural order within intact living specimens. As a result, a key blind spot remains: contaminant stress can simultaneously reshape behavior, disrupt internal structural organization, and destabilize physiological regulation, but these manifestations are rarely resolved within a unified observational framework.

Here, we address this gap by establishing Polarization Holographic Imaging Microscopy (PHIM) as a label‐free multiscale phenotyping framework for complex living aquatic organisms. Rather than serving merely as a tracking tool, PHIM is designed as an integrated measurement framework that combines polarization‐sensitive holographic imaging with computational volumetric reconstruction to access complementary phenotypic layers in intact organisms under native seawater conditions. This framework enables continuous four‐dimensional (4D) characterization of locomotor dynamics together with polarization‐resolved evaluation of endogenous structural organization, without mechanical scanning. In this way, PHIM extends volumetric holographic observation beyond motion alone by linking external behavioral dynamics with internal biophysical organization. More broadly, PHIM provides a non‐invasive and non‐destructive route to resolve sublethal functional perturbations in living organisms, extending contaminant assessment beyond static endpoint readouts toward dynamic phenotyping of intact biological function.

We use a nanoplastic–marine rotifer system as a high‐value demonstration of this framework. Marine rotifers (*Brachionus plicatilis*) are ecologically relevant, freely swimming zooplankton whose rapid locomotor dynamics and sensitivity to environmental stress make them particularly suitable for resolving subtle contaminant‐induced perturbations [28]. Within this model system, we test the hypothesis that nanoplastic exposure does not simply suppress motility, but instead manifests as coordinated functional reorganization across behavior, endogenous structural order, and physiological state. Using PHIM, we show that exposure to model polystyrene (PS) nanoplastics drives redistribution of organisms into distinct locomotor regimes rather than producing a uniform decline in activity, revealing altered exploration, reorientation dynamics, and behavioral phenotypes. This locomotor reorganization is accompanied by a reduced vesicle‐associated polarization anisotropy, while fluorescence‐based calcium imaging provides an orthogonal physiological correlate of altered intracellular signaling. By resolving organism‐scale locomotion and endogenous polarization phenotypes in intact living organisms, and integrating these label‐free PHIM readouts with fluorescence‐based validation, this work positions polarization holographic 4D phenotyping as a scalable and sensitive platform for multiscale assessment of contaminant‐induced dysfunction in aquatic systems.

## 2 Results

### 2.1 PHIM Enables Label‐Free 4D Phenotyping of Zooplankton in Natural Seawater

Directly resolving contaminant‐induced dysfunction in living aquatic organisms remains challenging as natural seawater is optically complex, with suspended particulates, multiple scattering, and a limited depth of field that confound conventional microscopy. In particular, widely used imaging assays project vertical motion onto a 2D plane and therefore fail to capture the full spatiotemporal structure of locomotor behavior in freely swimming organisms [7, 13]. To overcome these limitations, we established PHIM as a framework specifically designed for continuous label‐free 4D phenotyping in optically complex aquatic samples (Figure 1a). Unlike common imaging workflows that require fluorescent labeling or axial scanning, PHIM operates without chemical staining or mechanical sectioning, thereby preserving the natural optical and ecological context of the sample (Figure 1b).

**FIGURE 1:**
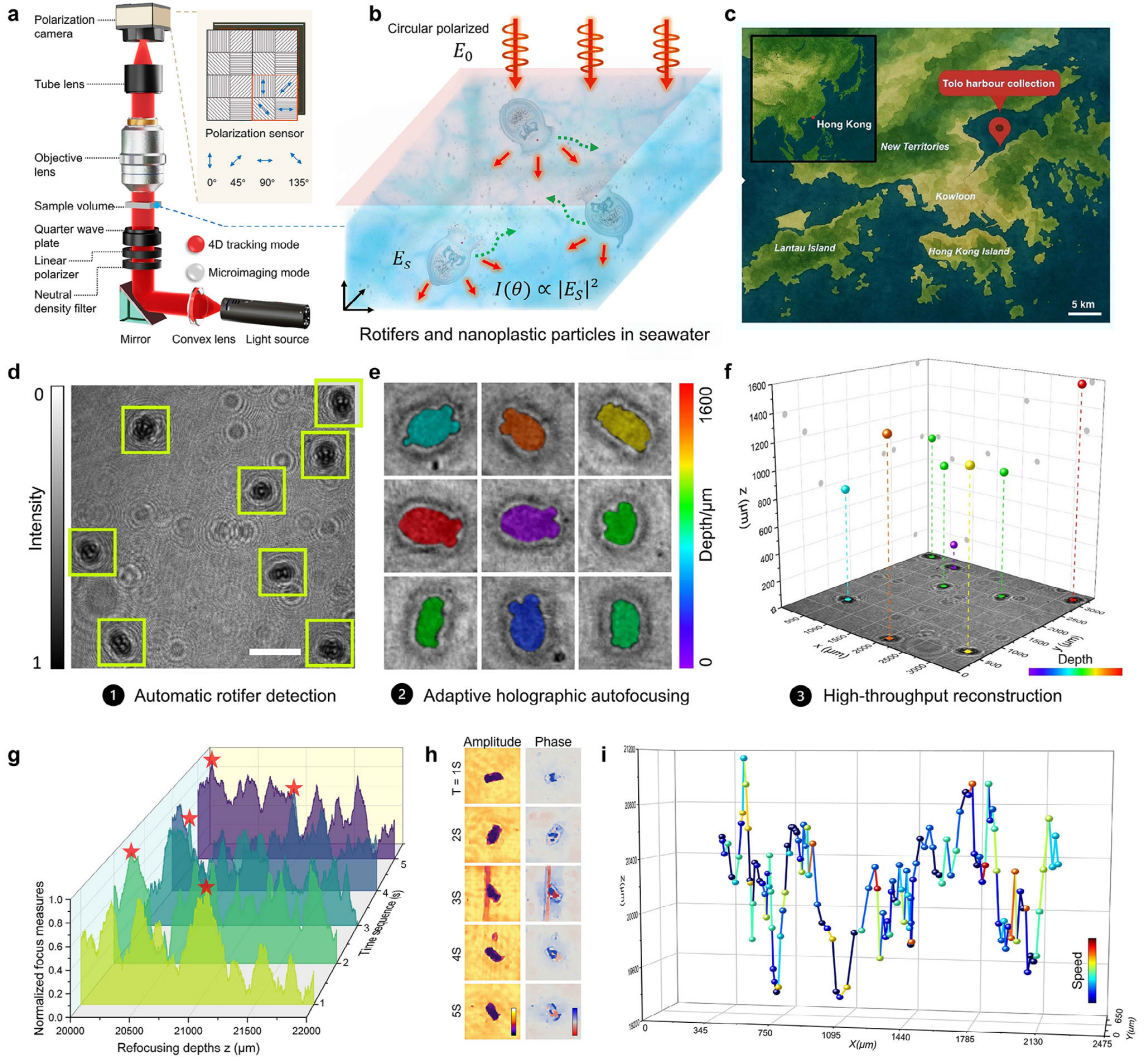
PHIM system and computational workflow for label‐free 4D phenotyping. (a) Optical configuration of PHIM. A circularly polarized laser illuminates the sample volume, and a polarization camera continuously records the holograms across four orientations. (b) Illumination traverses an aquatic volume containing marine rotifers and suspended particulates. The system encodes spatiotemporal coordinates and polarimetric signatures. (c) Field collection site at Tolo Harbour, Hong Kong, where natural seawater containing rotifer populations was acquired. (d–f) The high‐throughput computational reconstruction pipeline. (d) Deep‐learning‐based detection isolates 8 rotifers from a representative hologram. Scale bar: 500μm. (e) Adaptive autofocusing retrieves the precise axial position of 9 examples. (f) 3‐D point‐cloud rendering, visualizing the spatial distribution of detected individuals color‐coded by depth. (g–i) Evaluation of tracking fidelity. (g) Composite focusing curves identify global axial maxima (red stars), overcoming multimodal scattering interference typical of biological media. (h) Retrieved amplitude and phase images maintain morphological fidelity even at measurement boundaries (T = 3s). (i) A representative 4D trajectory reconstructed from 300 consecutive frames, capturing fine‐scale motility dynamics.

Using marine rotifers as a representative zooplankton model [28], PHIM achieved continuous volumetric observation in seawater collected directly from Tolo Harbour (Figure 1c) without filtration or purification. This configuration retained the suspended particulates and optical heterogeneity of the native environment rather than simplifying the medium to fit into an ideal laboratory background. Within this challenging setting, polarization‐resolved holographic imaging suppressed a substantial fraction of the multiple‐scattering background while preserving morphological visibility of intact organisms, including the ciliary corona and internal digestive structures in microimaging mode (Figure S1). This capability extends beyond standard label‐free tracking approaches, which primarily report organism position and gross morphology [25], by adding access to polarization‐derived readouts of endogenous structural organization. As a result, PHIM provides a measurement space in which locomotor dynamics and internal biophysical organization can be phenotyped within the same label‐free framework.

Computational volumetric reconstruction further revealed that marine rotifer swimming in seawater contains pronounced vertical dynamics (Figure 1d–i) that are not recoverable from planar observation alone [12, 29]. Individual trajectories exhibited rapid depth excursions, oscillatory movements, and abrupt directional changes across the full observation volume, demonstrating that a substantial fraction of natural locomotor behavior unfolds outside the focal plane used in conventional 2D assays [25, 30]. Consistent with this, trajectory descriptors derived from 2D projections underestimated both path length and reorientation frequency relative to full 3D reconstructions. These findings establish that the measurement limitation is not merely one of image quality, but of behavioral observability: planar assays systematically miss a component of organismal function that becomes critical when subtle stress‐induced perturbations must be detected before pronounced toxic effects emerge.

To translate the system design into a practical phenotyping workflow, we developed a high‐throughput reconstruction pipeline for dense scenes containing 20–50 organisms per frame. A lightweight YOLO11n detector first localized individual rotifers and defined regions of interest (ROIs) in the raw holograms (Figure 1d), achieving high detection accuracy with an F1 score of 0.954 and a ${\mathrm{mAP}}_{50}$ of 0.986 (Figures S2 and S3; Table S1). The network was used exclusively for rapid target localization before numerical reconstruction. For routine 4D tracking, each ROI was numerically back‐propagated over a calibrated axial search window of 3000μm at 100μm intervals, yielding 31 candidate planes per ROI per frame. Axial positions were determined using adaptive multimetric holographic autofocusing (AMHA) algorithm (Figure 1e,f and Note S1), and focal‐plane selection relied on the amplitude‐based AMHA score (Figure 1g). The 100μm interval was selected through step‐size sensitivity testing to balance focus stability and computational throughput. The composite metric provided more consistent focal‐plane identification than the individual focus metrics across all exposure conditions (Figures S4 and S5). The twin‐image is suppressed at the AMHA‐determined focal plane [31], and the reconstructed phase maps were retained only as an auxiliary quality‐control output (Figure 1h; Figure S6). The recovered 3D coordinates were subsequently linked across frames to generate continuous 4D trajectories, from which all behavioral phenotypes were derived (Figure 1i). In the benchmarked implementation, ROI‐level depth reconstruction required approximately 0.17s per organism, corresponding to a processing capacity exceeding $10^{4}$ ROI reconstructions per hour. Together, this workflow establishes PHIM as a high‐throughput, label‐free, and non‐destructive platform for volumetrically resolved phenotyping of freely swimming zooplankton in natural seawater.

### 2.2 Nanoplastic Stress Drives Functional Reorganization Rather Than Simple Motility Suppression

To determine whether nanoplastic exposure simply attenuates movement or instead reshapes locomotor function, we analyzed 4D swimming trajectories of marine rotifers under control conditions and after exposure to PS nanoplastics (Figures S7– S9; Table S2) of 500nm, 100nm, and 20nm (Figure S10). Representative trajectories revealed that the effect of exposure was not a uniform reduction in motion amplitude (Figure 2a). Control rotifers displayed extended exploratory paths with sustained displacement and moderate turning, consistent with unconstrained planktonic swimming [32, 33, 34]. By contrast, exposed groups diverged into distinct locomotor regimes. The 500nm condition largely preserved exploratory structure with a modest reduction in trajectory breadth, whereas the 100nm condition produced densely folded paths marked by frequent reorientation and localized activity. The 20nm condition, in turn, generated shortened trajectories with markedly constrained spatial coverage, indicating a shift toward spatially restricted locomotion rather than simple hypoactivity. These observations already suggested that nanoplastic stress acts by redistributing behavioral output into different movement modes, rather than by imposing a single monotonic suppression of motility.

**FIGURE 2:**
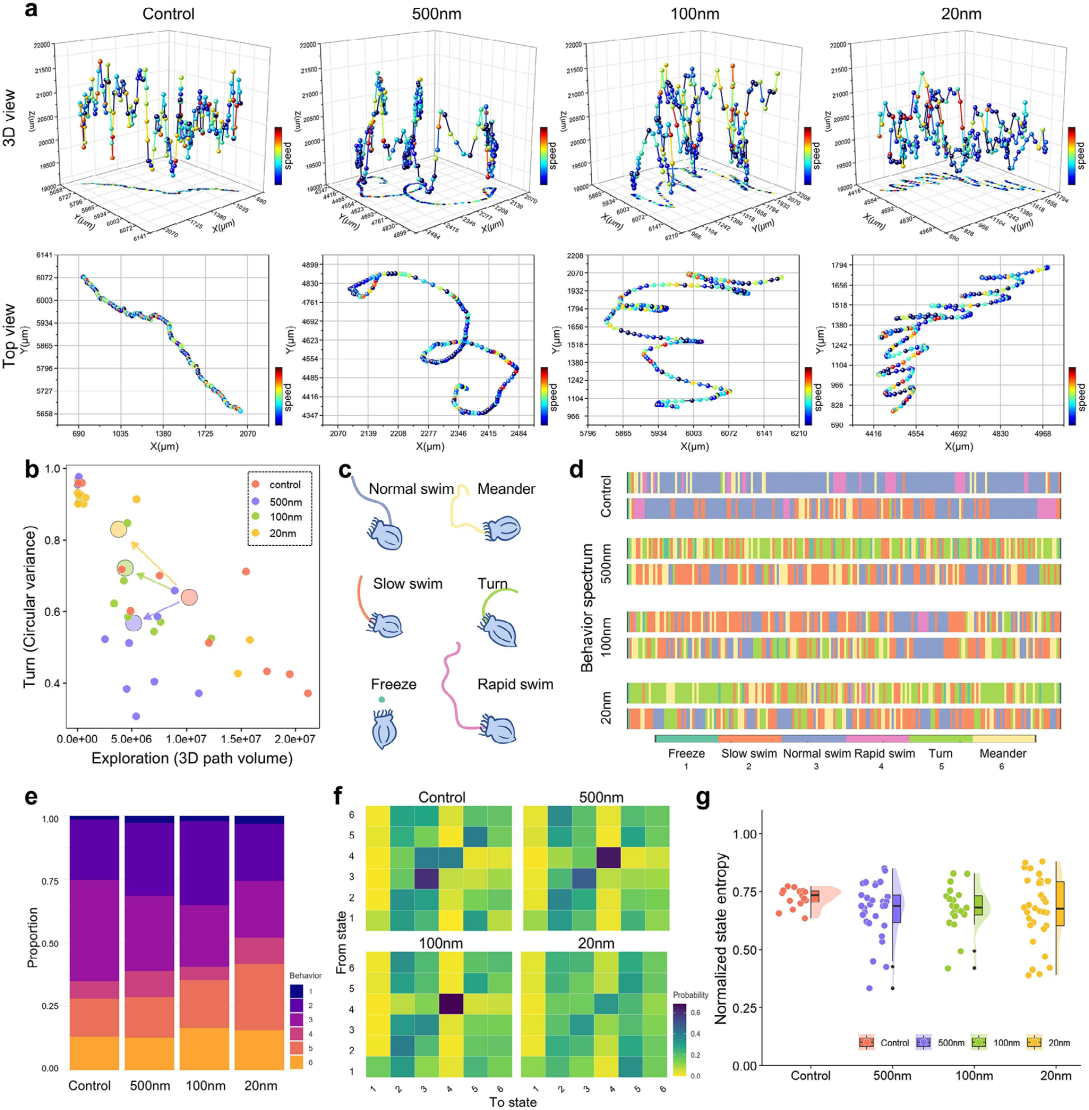
Nanoplastic stress drives functional reorganization of locomotor behavior in marine rotifers. (a) Representative 4D swimming trajectories and corresponding top‐view projections of individual rotifers under control conditions and exposure to 500nm, 100nm, and 20nm PS nanoplastics. Trajectories are color‐coded by instantaneous speed. (b) Exploration‐turning behavioral state space, defined by 3D path volume and circular variance. Points denote individual trajectories, and arrows indicate centroid shifts relative to control. (c) Canonical locomotor motifs used for trajectory segmentation. (d) Temporal behavioral spectra of 8 representative trajectories showing the time‐resolved sequence of locomotor states 1–6. (e) Population‐level behavioral state occupancy calculated from all valid trajectories, expressed as the proportion of total trajectory time spent in each locomotor state across conditions. (f) State‐to‐state transition probability matrices summarizing behavioral dynamics, highlighting distinct transition structures across conditions. (g) Normalized behavioral state entropy, reflecting state‐use diversity and evenness. Quantitative analyzes were performed using N=3 independent biological replicates; sample sizes were control, n=17; 500nm, n=28; 100nm, n=20; and 20nm, n=30 valid single‐organism trajectories.

We next quantified these trajectory‐level differences in an exploration‐turning state space defined by 3D path volume and circular variance (Figure 2b; Note S2). All exposed groups deviated from the control centroid, but critically, they did so along different directions rather than along a common axis of declining activity. The 500nm group showed only a mild displacement toward reduced exploration and turning variability. In contrast, the 100nm group shifted predominantly along the turning axis while largely retaining exploratory range, consistent with an erratic, highly reorienting locomotor program. The 20nm group exhibited the strongest contraction in exploration together with increased turning statistics, indicating confinement to a more spatially restricted regime. This directional separation demonstrates that decreasing particle size partitions behavior into distinct locomotor fates, revealing a non‐linear restructuring of state space rather than a graded weakening of movement.

To resolve the temporal architecture underlying these divergent locomotor regimes, continuous trajectories were segmented into six canonical behavioral motifs: freeze, slow swim, normal swim, rapid swim, turn, and meander according to instantaneous speed and the turning angle (Figure 2c; Table S3). Figure 2d,e provides complementary views of this state‐based analysis. The temporal behavioral spectra in Figure 2d show two representative trajectories per condition and preserve the ordering, persistence, and switching of behavioral states over time. In contrast, the stacked occupancy plot in Figure 2e summarizes the population‐level time budget of each state across all valid single‐organism trajectories (N=3 biological replicates; n=95 trajectories in total). Population‐level occupancy confirmed a redistribution of behavioral time budgets. Relative to controls, the 500nm and 100nm groups spent more time in rapid and slow swimming states, whereas the 20nm group exhibited increased occupancy of turning and freeze states, indicating a shift away from sustained exploratory swimming. Consistent with this state redistribution, transition‐probability matrices revealed altered state‐switching architectures after nanoplastic exposure (Figure 2f). Control animals exhibited relatively balanced switching with frequent returns to normal swimming, while exposed groups showed increasingly asymmetric transitions, including strengthened transitions into rapid locomotor states under 500 and 100nm exposure and impaired recovery to sustained swimming under 20nm exposure. Together, these results indicate that nanoplastic stress reconfigures not only where organisms move, but also how locomotor states are sequenced and maintained over time.

As a compact descriptor of behavioral state diversity, normalized behavioral state entropy revealed exposure‐dependent changes in how rotifers distributed time across the six locomotor states (Figure 2g). Rather than being interpreted as a simple marker of increased or decreased activity, entropy summarizes whether behavior is concentrated within a narrow set of states or distributed across a broader state repertoire. A reduction in entropy indicates dominance by fewer locomotor states, whereas an increase may reflect more fragmented or dispersed state usage. Therefore, entropy was interpreted together with state occupancy and transition‐probability matrices to distinguish constrained behavioral repertoires from altered state‐switching organization. Importantly, the entropy changes did not simply mirror movement magnitude. Instead, they captured a redistribution of behavioral time budgets within a reorganized locomotor landscape [35, 36]. Together with the exploration–turning state space, occupancy profiles, and transition matrices, these results show that nanoplastic exposure does not merely suppress rotifer motility, but reshapes locomotor organization into distinct functional phenotypes. This state‐space perspective reframes nanoplastic toxicity from gross activity loss to altered behavioral organization, providing the first phenotypic layer of the multiscale dysfunction resolved by PHIM.

### 2.3 Integrated Behavioral Phenotyping Resolves Coordinated Functional Redistribution

While the 4D state space analysis established that nanoplastic exposure reshaped locomotor dynamics, individual kinematic descriptors alone were insufficient to explain how organismal function was redistributed under stress. In freely swimming organisms, behavioral adaptation is not encoded by a single variable, but emerges from coupled changes in exploration, directional persistence, reorientation, activity, and temporal regularity [37]. We therefore next investigated whether the observed locomotor divergence could be reformulated as an integrated functional phenotype rather than a series of isolated trajectory abnormalities. To this end, we organized 27 trajectory‐derived descriptors (Note S2 and Figure S11) into five ecological functional domains [34, 38], including space use, directional persistence, reorientation dynamics, activity intensity, and behavioral regularity, thereby converting high‐dimensional locomotor data into a domain‐resolved phenotyping framework (Figure 3a).

**FIGURE 3:**
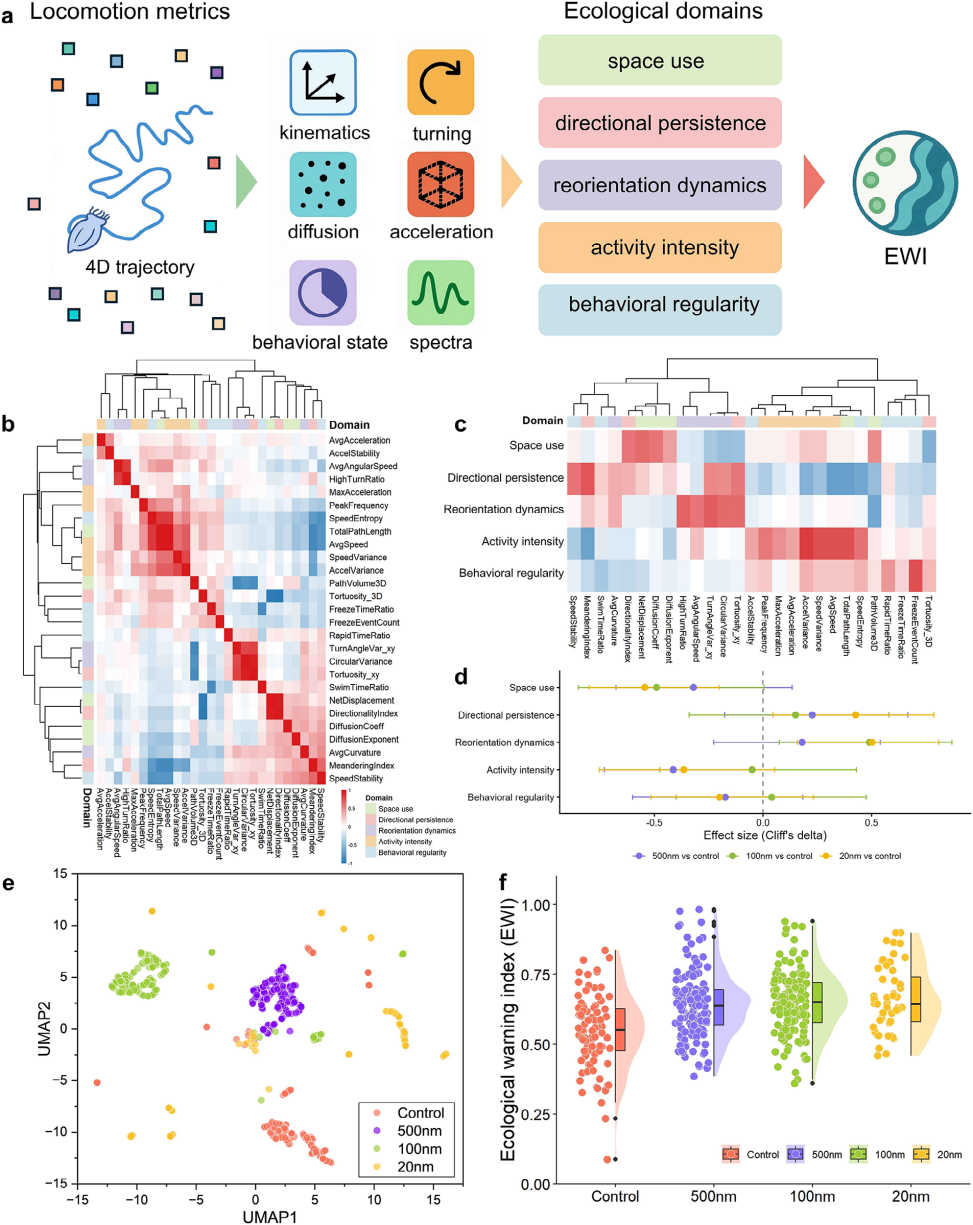
High‐dimensional behavioral phenotyping reveals coordinated ecological functional domains. (a) Workflow for organizing 27 trajectory descriptors into five ecological domains and integrating them into the ecological warning index (EWI). (b) Spearman correlation matrix of the 27 locomotor descriptors, showing domain‐level clustering. (c) Correlations between individual descriptors and ecological domain scores, validating the domain assignment. (d) Domain‐level effect sizes relative to control, shown as Cliff's delta with bootstrap 95% confidence intervals. (e) UMAP visualization of individual behavioral profiles based on ecological domain scores, visualizing condition‐dependent separation in functional space. (f) Distribution of EWI across conditions, summarizing integrated functional deviation from control. Quantitative analyzes were performed using N=3 independent biological replicates; sample sizes were control, n=162; 500nm, n=207; 100nm, n=190; and 20nm, n=107 valid single‐organism trajectories.

The multidomain behavioral analysis included n=666 valid single‐organism trajectories from N=3 independent biological replicates per condition (Table S4). This integrated representation revealed that nanoplastic perturbation was highly structured rather than diffusely distributed across all behavioral variables (Figure 3b). Metrics within the same domain showed coherent covariation, whereas exposure‐dependent shifts emerged as selective redistributions across functional axes (Figure 3c). Relative to control animals, the 500nm group exhibited only limited displacement, indicating that the global behavioral program remained largely preserved despite mild perturbation (Figure 3d). In contrast, the 100nm group showed a pronounced shift centered on reorientation‐related features, consistent with the densely folded and highly redirected trajectories observed in the 4D reconstruction. The 20nm group displayed the broadest phenotypic redistribution, characterized by reduced space use, loss of behavioral regularity, and strong deviation in reorientation‐associated descriptors, indicating a transition toward a more spatially constrained and less adaptable functional state. These results show that decreasing particle size does not simply intensify one common defect, but progressively reorganizes behavior into distinct functional modes.

The coordinated nature of this functional redistribution was further visualized by projecting individual behavioral profiles into a low‐dimensional phenotype space using UMAP (Figure 3e). UMAP was used for visualization, whereas statistical comparisons were performed on single‐trajectory domain scores. Control rotifers occupied a relatively compact region, consistent with coordinated baseline organization across exploration, directional persistence, reorientation, activity, and behavioral regularity. In contrast, nanoplastic‐exposed groups diverged from this control‐defined state in distinct directions rather than shifting along a single monotonic axis. The 100nm group separated along a phenotype associated with enhanced reorientation and localized activity, whereas the 20nm group occupied a more distant region characterized by restricted space use and altered behavioral regularity. Thus, exposure did not simply increase behavioral variability, but redistributed individuals across interpretable functional phenotypes.

To summarize these multidomain deviations in a compact form, we computed an ecological warning index (EWI) relative to the control‐defined functional state (Figure 3f). EWI values shifted upward after nanoplastic exposure, with the strongest deviation observed in the 20nm group, consistent with its broader displacement in domain‐level effect sizes and phenotype space. Importantly, EWI was not intended to replace the multidimensional behavioral analysis with a single score; rather, it provides a concise summary of coordinated deviation across multiple ecological functions. Together, these results show that nanoplastic stress reorganizes locomotion into distinct, interpretable functional phenotypes, converting behavioral divergence into a scalable readout of contaminant‐induced dysfunction.

In addition to the particle‐size comparison at a fixed mass concentration, we performed a concentration‐sensitivity validation using 20nm PS nanoplastics at 0.2, 1, and $5\;\mathrm{mg}\;L^{-1}$, together with a control group (Figure S12). The lower‐concentration groups showed weaker phenotypic shifts than the $5\;\mathrm{mg}\;L^{-1}$ group, supporting the concentration sensitivity of PHIM‐resolved behavioral dysfunction while indicating that the added experiment serves as a targeted concentration‐sensitivity validation rather than a complete dose‐response model.

### 2.4 Polarization Phenotyping Reveals Endogenous Structural Disorder Associated With Nanoplastic Accumulation

Having established that nanoplastic exposure redistributes rotifers into distinct functional behavioral phenotypes, we next investigated whether this locomotor reorganization was accompanied by changes in internal structural organization. Motion readouts alone cannot address this question. A central advantage of PHIM is that it extends label‐free volumetric phenotyping beyond kinematics by providing polarization‐sensitive access to endogenous structural order within intact organisms. In addition to the high‐throughput 4D holographic tracking mode used for locomotor trajectory reconstruction, PHIM incorporates a high‐resolution microimaging mode enabled by a high‐numerical‐aperture imaging module (Table S5). We therefore used PHIM polarization contrast to probe digestive compartments, where nanoplastic accumulation may exert early biophysical effects.

To ensure that the observed fine polarization phenotype did not arise from imaging artifacts, we performed a series of calibration and control analyzes. The polarization camera was calibrated using known linear polarization states, confirming accurate angle of polarization (AoP) recovery and stable degree of linear polarization (DoLP) measurements (Figure S13 and Table S6). Additional defocus and orientation controls showed that vesicle‐associated polarization patterns were qualitatively robust to modest focus and specimen‐orientation perturbations (Figure S14).

In control rotifers, polarization mapping revealed organized AoP textures and elevated DoLP contrast in digestive vesicle‐associated regions (Figure 4a), with similar polarization signatures observed in *Daphnia* (Figure S15). These patterns are consistent with preserved endogenous anisotropic organization in digestive compartments. By contrast, nanoplastic‐exposed rotifers showed reduced vesicle‐associated DoLP and disrupted AoP spatial organization. At the single‐vesicle level, radial AoP and DoLP profiles further showed attenuation of the structured polarization patterns observed in control vesicles (Figure 4b). Thus, the polarization readout reports an internal optical phenotype that complements the locomotor phenotypes.

**FIGURE 4:**
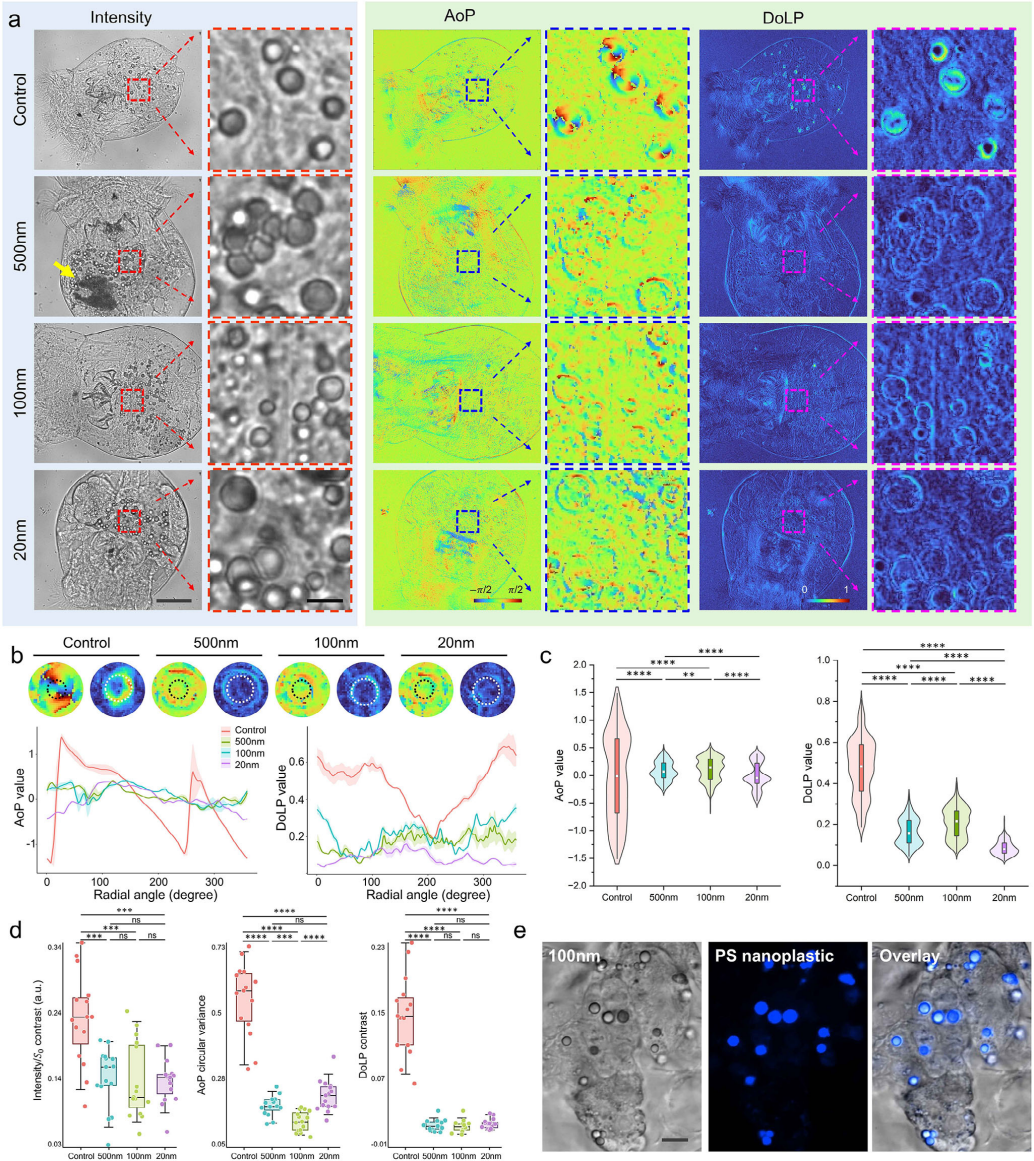
Nanoplastic exposure is associated with altered vesicle‐associated polarization phenotypes in marine rotifers. (a) Representative intensity, angle of polarization (AoP), and degree of linear polarization (DoLP) maps of rotifers across exposure conditions. Control rotifers show organized vesicle‐associated AoP textures and elevated DoLP contrast, whereas nanoplastic‐exposed rotifers exhibit disrupted AoP organization and reduced DoLP. Scale bars: 30μm (main), 5μm (inset). (b) Single‐vesicle polarimetric profiling showing radial AoP and DoLP distributions. Nanoplastic exposure weakens the structured polarization patterns observed in control vesicles. (c) Distributions of AoP and DoLP values across exposure conditions, showing reduced polarization dynamic range after nanoplastic exposure. (d) Rotifer‐level quantification of intensity contrast, AoP circular variance, and DoLP contrast. Each point represents the mean of three vesicle ROIs from one rotifer (n=15 rotifers per condition; N=3 independent biological replicates). (e) Confocal fluorescence validation showing fluorescent nanoplastic signals within internal digestive regions and their spatial association with regions of reduced vesicle‐associated polarization contrast. Scale bars: 10μm.

Quantitative analysis supported this exposure‐associated polarization phenotype. Relative to controls, nanoplastic‐exposed groups showed compressed AoP and DoLP distributions, consistent with a broad reduction in polarization contrast (Figure 4c). We further quantified intensity contrast, AoP circular variance, and DoLP contrast at the rotifer level by averaging three vesicle ROIs within each organism (Figure 4d; Figure S16). This analysis included n=60 rotifers from N=3 independent biological replicates. To test whether polarization features provided information beyond intensity or morphology alone, we performed an organism‐grouped feature‐ablation analysis using descriptors extracted from the same vesicle ROIs. Adding polarization‐derived features improved exposure discrimination relative to $S_{0}$ intensity‐only descriptors (Figure S17), indicating that polarization captures complementary information not fully explained by intensity contrast.

We then examined whether the polarization phenotype was spatially associated with particle burden. Confocal fluorescence imaging showed fluorescent PS signals mainly within internal digestive regions of exposed rotifers, whereas controls showed negligible background fluorescence (Figure S18). Regions with stronger fluorescent nanoplastic burden spatially corresponded to regions with reduced vesicle‐associated polarization contrast (Figure 4e). Importantly, we do not interpret the AoP and DoLP maps as direct label‐free identification of individual nanoplastic particles. Particle identity, morphology, and in‐organism localization were validated using orthogonal characterization and fluorescence measurements. Likewise, the reduced DoLP and disrupted AoP spatial coherence should be interpreted as an exposure‐associated polarization phenotype rather than direct proof of structural collapse, because scattering, depolarization, and compartment geometry may also contribute to the observed changes. Within these boundaries, the polarization measurements provide a second phenotypic layer of PHIM‐resolved dysfunction, linking organism‐scale locomotor reorganization to altered internal optical organization under nanoplastic exposure.

### 2.5 Calcium Imaging Provides an Orthogonal Physiological Correlate of Nanoplastic‐Induced Dysfunction

To further contextualize the locomotor and polarization phenotypes, we next examined whether nanoplastic exposure was accompanied by altered intracellular calcium activity. Because behavioral reorganization and polarization‐derived internal optical phenotypes do not directly report physiological signaling, we used Fluo‐4 AM calcium imaging as an orthogonal fluorescence‐based readout of physiological activity (Figure 5a,b). Intracellular calcium is closely associated with excitability, contractility, and stress‐related signaling, making it a useful indicator of physiological perturbation in living rotifers [39].

**FIGURE 5:**
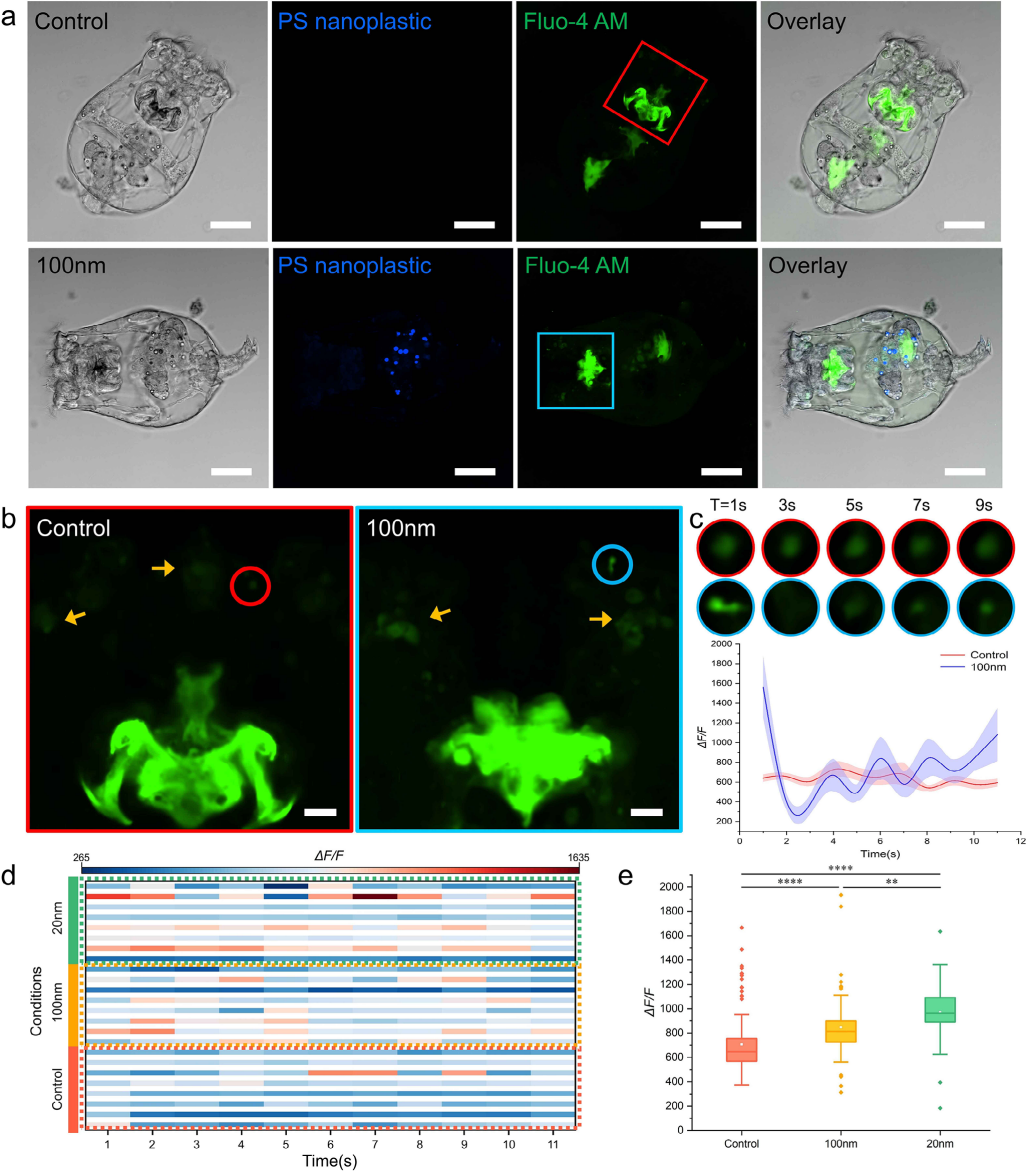
Fluorescence‐based calcium imaging reveals physiological perturbations associated with nanoplastic exposure. (a) Representative confocal images showing brightfield morphology, fluorescent PS nanoplastics (blue), Fluo‐4 AM calcium signals (green), and merged overlays in control and 100nm nanoplastic‐exposed rotifers. Colored boxes mark regions selected for enlarged and time‐lapse analysis. Scale bars: 50μm. (b) Enlarged Fluo‐4 AM images of selected cephalic regions in control and 100nm nanoplastic‐exposed rotifers. Arrows and circles indicate representative calcium‐positive regions. Scale bars: 10μm. (c) Time‐lapse imaging of selected regions showing representative calcium‐signal dynamics. Corresponding ΔF/F traces indicate larger and less stable calcium transients after nanoplastic exposure. (d) Heatmaps of temporal calcium fluorescence across individual rotifers in the control, 100nm, and 20nm groups. (e) Quantification of peak ΔF/F, showing elevated calcium‐response amplitudes in nanoplastic‐exposed rotifers.

In control rotifers, Fluo‐4 AM signals were spatially localized to anatomically active regions, including the mastax and cerebral ganglion‐associated areas, consistent with organized baseline physiological activity in intact animals. In contrast, nanoplastic‐exposed rotifers showed altered calcium‐signal distribution together with fluorescent PS nanoplastic signals (Figure 5a). Enlarged views of selected regions further revealed brighter and more spatially heterogeneous calcium‐positive regions after 100nm nanoplastic exposure (Figure 5b). Time‐lapse imaging and corresponding ΔF/F traces showed that exposed rotifers exhibited larger and less stable calcium transients relative to controls, indicating altered calcium‐signal dynamics after exposure (Figure 5c).

Population‐level analysis supported this observation. Heatmaps of temporal fluorescence revealed stronger and more heterogeneous calcium responses in nanoplastic‐exposed groups compared with controls (Figure 5d). Quantification of peak ΔF/F further showed elevated calcium‐response amplitudes in both the 100nm and 20nm exposure groups (Figure 5e). These changes paralleled the multiscale phenotypes observed above: the 100nm group, which showed enhanced reorientation and localized activity, also exhibited fluctuating calcium responses, whereas the 20nm group showed higher peak calcium responses together with stronger spatially constrained locomotor alterations.

Importantly, these calcium measurements do not establish a causal sequence linking nanoplastic burden, polarization changes, calcium signaling, and behavior. Rather, they provide an independent physiological correlate showing that the behavioral and internal optical phenotypes resolved by PHIM occur in parallel with altered intracellular signaling. Together, the 4D locomotor, polarization, and calcium readouts indicate that nanoplastic exposure is associated with coordinated dysfunction across organismal, internal optical, and physiological levels, rather than a simple reduction in motility alone.

## 3 Discussion

In this work, we establish PHIM as a multiscale phenotyping framework for resolving contaminant‐induced dysfunction in living aquatic organisms. Using a nanoplastic exposure–marine rotifer system as a high‐value demonstration, we show that nanoplastic stress does not simply suppress locomotion, but instead manifests as coordinated functional reorganization across 4D locomotor behavior, endogenous structural anisotropy, and fluorescence‐based physiological states. The central contribution of PHIM is therefore not merely an imaging platform with improved visualization capability, but the ability to convert dynamic organismal responses into quantitative, non‐destructive phenotypes in complex living systems.

A major biological implication of this study is that nanoplastic exposure should not be interpreted solely as a uniform reduction of activity. The dominant response resolved here was not monotonic suppression, but state redistribution [40, 41]. Exposed marine rotifers diverged into distinct locomotor regimes with altered exploration, reorientation structure, transition dynamics, and behavioral phenotypes. This distinction is conceptually important because average speed endpoint impairment (Table S7) can obscure how animals reallocate their behavioral time budgets under stress. Organisms with similar activity levels may differ substantially in whether they sustain exploratory swimming, repeatedly reorient, or fragment behavior across multiple states [12, 13]. By resolving these differences in 4D, PHIM reframes nanoplastic‐induced behavioral toxicity from a scalar loss of movement to a reorganization of locomotor state space. This perspective is particularly relevant for sublethal contaminant assessment, where functional changes may appear before severe endpoint phenotypes become dominant [10, 11].

The methodological contribution of PHIM is the access to multiscale living phenotypes. Conventional biochemical and molecular assays provide valuable mechanistic information, but they are often destructive, endpoint‐oriented, population‐averaged, or disconnected from the intact dynamics of living individuals [14, 42]. Spectroscopic [18, 19] and fluorescence‐based approaches [23, 24] offer chemical specificity and labeled localization, but are less suited for continuous, label‐free, volumetric phenotyping of freely behaving organisms in realistic aquatic environments. PHIM addresses this gap by integrating polarization‐sensitive holography with computational volumetric reconstruction, enabling 4D locomotor phenotyping together with polarization‐derived assessment of endogenous internal optical organization under seawater conditions.

It is also important to define the optical scope of PHIM precisely. PHIM does not provide nanoscale optical resolution and should not be interpreted as a label‐free method for directly visualizing individual nanoplastic particles. In this study, the core PHIM readouts are label‐free 4D locomotor trajectories and polarization‐derived internal optical phenotypes. By contrast, particle identity, morphology, chemical composition, and in‐organism localization were independently assessed using SEM/TEM, O‐PTIR, and fluorescence/confocal imaging [28]. This distinction is essential because the strength of PHIM is not to replace nanoscale particle‐characterization methods, but to connect nanoplastic exposure with organism‐scale and internal phenotypic consequences in intact living animals.

The particle‐size comparison should also be interpreted within the exposure‐metric framework of the study. The main experiments compared 20, 100, and 500nm PS nanoplastics at a fixed mass concentration of $5\;\mathrm{mg}\;L^{-1}$, selected as a benchmark challenge exposure that enabled detectable particle uptake, sufficient freely swimming organisms, and measurable sublethal phenotypes. Equal mass concentrations, however, do not correspond to equal particle‐number or surface‐area burdens across sizes. At the same mass concentration, smaller particles impose substantially higher particle‐number and surface‐area burdens [3, 43], which may influence uptake probability, biological interaction, aggregation behavior, and surface‐mediated effects (Table S2). Thus, the results are best described as particle‐size‐associated responses under fixed mass‐normalized exposure, rather than as a diameter‐only toxicity effect.

Several limitations define the scope of the present study. First, the experiments used model fluorescent PS nanoplastics, whereas environmentally relevant particles are heterogeneous in composition, morphology, surface chemistry, weathering state, aggregation behavior, and co‐contaminant context. Second, the benchmark exposure is useful for controlled phenotyping but is higher than many reported environmental concentrations (Table S8); future work should extend PHIM to chronic, low‐dose, and environmentally aged particles. Third, although behavioral, polarization, and calcium readouts changed in a coordinated manner, the present study does not establish their causal hierarchy. Time‐resolved exposure experiments, perturbation of particle uptake, independent modulation of calcium signaling, and additional structural or biochemical assays will be needed to test mechanistic ordering. Within these boundaries, the present study establishes PHIM as a scalable and physiologically contextualized platform for resolving contaminant‐induced dysfunction in living aquatic organisms.

## 4 Experimental Section

### 4.1 Optical Configuration of PHIM

The PHIM system integrates snapshot polarization imaging with in‐line digital holography for multiscale phenotyping of living aquatic organisms (Figure 1a). The system was operated in two imaging modes according to the required field of view, throughput, and spatial resolution. For high‐throughput 4D tracking, a 632.8nm laser beam was collimated and expanded by a convex lens (f=75mm), and then passed through a linear polarizer and a quarter‐wave plate to generate circularly polarized illumination. The beam illuminated the sample volume in transmission. In the lens‐free implementation used for large‐field rotifer tracking, the transmitted and diffracted wavefronts propagated directly to a polarization‐resolved CMOS sensor (PHX050S1‐P/Q, Lucid Vision Labs, Canada). When higher lateral sampling was required, a low‐magnification objective–tube lens module could be inserted in the detection path. For high‐resolution polarization microimaging, the coherent laser source was replaced by a broadband halogen lamp (Philips 7724) to reduce coherent speckle, and an objective–tube lens module was used before the same polarization camera. This mode used a high‐NA objective (Nikon S Plan Fluor 40×, NA 0.65) for fine morphological analysis and mapping of endogenous polarization organization. The measured lateral resolution in this mode was 2.19μm.

In both modes, the polarization camera contained a 2×2 micro‐polarizer array that simultaneously recorded $0^{\circ }$, $45^{\circ }$, $90^{\circ }$, and $135^{\circ }$ polarization channels over a 2448×2048 pixel grid with a sensor subpixel size of 3.45μm. The snapshot acquisition of the four polarization channels avoids motion artifacts associated with sequential polarization measurements. Because a complete polarization measurement requires one 2×2 superpixel, the effective polarimetric sampling is 6.90/Mμm, where M is the effective system magnification. Thus, the polarization camera does not change the diffraction‐limited optical resolution set by the imaging geometry or objective NA, but it reduces the native sampling density of full polarization maps by a factor of two in each lateral direction. Technical specifications of the 4D tracking and high‐resolution microimaging modes, including field of view, lateral resolution, axial localization precision, object‐plane pixel size, and effective polarimetric sampling, are summarized in Table S5.

### 4.2 Deep‐Learning‐Based ROI Detection

A lightweight deep‐learning detector was used to automatically localize organism ROIs from raw holographic intensity frames before numerical refocusing. We used the Ultralytics YOLO11n object detector and manually annotated individual rotifers with COCO‐style bounding boxes. The annotated dataset contained 600 holographic frames and 22,739 rotifer instances, which were divided into training, validation, and held‐out test subsets at a 70%/20%/10% ratio. The original 1224×1024 pixel frames were resized to 960×960 pixels for network training.

The detector was trained using AdamW optimization with an initial learning rate of $2.0\times 10^{-3}$, a batch size of 8, and 230 training epochs. Detection performance was evaluated on the held‐out test set using precision, recall, F1 score, ${\mathrm{mAP}}_{50}$, and ${\mathrm{mAP}}_{50:95}$, with detailed training and evaluation settings summarized in Table S1.

The deep‐learning model was used only for rapid ROI localization and cropping. It was not used to determine axial position, assign behavioral states, calculate the ecological warning index, or infer nanoplastic toxicity. After ROI detection, axial localization was performed by AMHA using physics‐based numerical propagation and amplitude‐based focus metrics, and all behavioral phenotypes were calculated from the reconstructed 4D trajectories.

### 4.3 Adaptive Multimetric Holographic Autofocusing (AMHA)

To robustly determine the true object plane for each ROI without manual tuning, we developed an AMHA strategy that combines angular‐spectrum back‐propagation with data‐driven weighting of complementary focus metrics. Each hologram U(x,y;0) was numerically propagated along the axial direction z using the angular‐spectrum method [44]:

(1) $$\begin{array}{c} U\left(x,y;z\right)=F^{-1}\;\left\{F\left\{U\left(x,y;0\right)\right\}\;\exp\left[i2\pi z\sqrt{\lambda^{-2}-f_{x}^{2}-f_{y}^{2}}\right]\right\}, \end{array}$$

where F denotes the 2‐D Fourier transform, λ the illumination wavelength, and $\left(f_{x},f_{y}\right)$ represents spatial frequency variables. Propagation was performed with micrometer‐scale axial steps to ensure accurate depth sampling. For each detected ROI, numerical refocusing was performed over a calibrated axial search range of 19000μm to 22000μm with a step size of 100μm, yielding 31 reconstructed amplitude images per organism per frame. The focal plane was selected as the depth maximizing the AMHA composite focus score.

For each reconstructed slice, three complementary sharpness metrics, Tenengrad gradient energy [45], Laplacian variance [46], and Brenner function [47], were computed on high‐pass‐filtered amplitude images. These metrics capture distinct aspects of image focus, including edge strength, fine‐scale texture, and grayscale transitions. Each metric was normalized across depth, and adaptive weights were assigned based on its dynamic range to form a composite focus score. The focal plane was identified as the depth maximizing this composite score, yielding a sharply focused reconstruction for downstream analysis. Detailed formulations and validation of AMHA are provided in Note S1.

Although angular‐spectrum propagation yields a complex reconstructed field containing both amplitude and phase, focal‐plane selection in this study was based on amplitude‐derived focus metrics. Because the in‐line holographic configuration can contain twin‐image artifacts, reconstructed phase distributions were not interpreted as absolute quantitative phase maps. To improve visualization quality, a constrained complex total variation (CCTV)‐based refinement was further applied at the AMHA‐determined focal plane [31].

### 4.4 Nanoplastic Characterization

To ensure that observed behavioral and polarization responses could be unambiguously attributed to particle size and polymer identity, fluorescent PS nanospheres with diameters of 20nm, 100nm and 500nm (Zhichuan Technology, China) were used as model nanoplastics. Uniform blue fluorescence was verified in the blue channel for all sizes. Particle morphology was characterized by scanning electron microscopy (SEM; S‐4800, Hitachi, Japan) and transmission electron microscopy (TEM; Talos F200S G2, Thermo Fisher Scientific, USA). Polymer identity and surface functionality were confirmed using optical photothermal infrared spectroscopy (O‐PTIR; mIRage, Photothermal Spectroscopy Corp., USA). Spectra were baseline‐corrected and smoothed with PTIR Studio v4.4 prior to analysis.

### 4.5 Rotifer Culture and Nanoplastic Exposure

Marine rotifers (*Brachionus plicatilis*) were collected from Tolo Harbour, Hong Kong (Coordinates: $22^{\circ }26^{\prime }50^{\prime \prime }N,114^{\circ }11^{\prime }17^{\prime \prime }E$) and maintained in natural seawater at 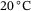 under a 12h:12h light: dark cycle. Cultures were fed *Chlorella pyrenoidosa*. For exposure assays, rotifers were adjusted to a density of $2\times 10^{4}\;\mathrm{ind}.L^{-1}$ and incubated with fluorescent PS nanoplastics at a concentration of $5\;mgL^{-1}$ (Table S2) for 48h. This concentration was selected as a benchmark challenge exposure to enable mass‐normalized comparison across particle sizes, ensure detectable particle uptake, and maintain sufficient freely swimming organisms for 4D trajectory reconstruction. Similar mg $L^{-1}$‐level exposure conditions have been used in previous studies involving rotifers and other aquatic organisms (Table S7).

Prior to imaging, rotifers were transferred to glass‐bottom dishes (diameter 35mm) and equilibrated for 1min. Volumetric datasets were acquired over a duration of 20s, yielding continuous 4D recordings of freely swimming rotifers.

### 4.6 4D Trajectory Reconstruction and Behavioral State Analysis

Multi‐target 4D trajectories were reconstructed over time by combining Kalman filtering with global assignment using the Hungarian algorithm. Detections in consecutive frames were linked using a linear state‐space motion model, and only tracks persisting for at least 100 frames were retained for downstream behavioral analysis.

For each valid trajectory, coordinates were sorted by frame and smoothed using a 5‐frame moving‐average filter. Instantaneous frame‐to‐frame centroid displacement speed and turning angle were then calculated. Reorientation‐dominated frames were assigned first: frames with turning angle $\theta >60^{\circ }$ were classified as “turn,” whereas frames with $30^{\circ }<\theta \leq 60^{\circ }$ were classified as “meander.” The remaining frames were classified according to swimming speed as freeze $\left(v<0.2\;\mathrm{pixel}\;{\mathrm{frame}}^{-1}\right)$, slow swim $\left(0.2\leq v<1.0\;\mathrm{pixel}\;{\mathrm{frame}}^{-1}\right)$, normal swim $\left(1.0\leq v<2.5\;\mathrm{pixel}\;{\mathrm{frame}}^{-1}\right)$, or rapid swim $\left(v\geq 2.5\;\mathrm{pixel}\;{\mathrm{frame}}^{-1}\right)$. Using the calibrated pixel size of 6.9 μm per pixel and the median frame interval of 0.127 s extracted from image timestamps, these speed thresholds correspond to approximately 10.9, 54.3, and 135.8 $\mu m\;s^{-1}$, respectively (Table S4). The same fixed thresholds were applied to all experimental groups.

For each valid single‐organism trajectory, we calculated behavioral‐state occupancy, state‐to‐state transition probabilities, and normalized Shannon entropy. For K=6 behavioral states, entropy was calculated as $H=-\sum_{i}p_{i}\log_{2}p_{i}$, where $p_{i}$ is the empirical probability of state i. The entropy was normalized by $\log_{2}K$, yielding a score from 0 to 1, where lower values indicate more stereotyped state usage and higher values indicate a more evenly distributed behavioral repertoire.

### 4.7 High‐Dimensional Behavioral Metrics and Ecological Domain Structure

To validate the categorization of the 27 behavioral metrics (P1–P27, Note S2) into five ecological functional domains, space use, directional persistence, reorientation dynamics, activity intensity, and behavioral regularity, we performed a data‐driven structural analysis. First, all metrics were standardized (Z‐scored) across individuals to normalize differences in units and scales. We then calculated pairwise Spearman rank correlations among all 27 metrics to assess their internal structure. Hierarchical clustering was applied to the correlation matrix using average linkage with a dissimilarity measure of 1−r to visualize natural groupings.

To further quantify the association between individual metrics and their hypothesized domains, we computed a composite domain score for each individual by averaging the Z‐scores of all constituent metrics within a given domain. We then evaluated the strength of the association between each metric and the five domain scores using Spearman correlation. This analysis confirmed that metrics exhibited strong, specific associations with their assigned ecological domains, supporting the functional validity of the proposed classification framework. All analyzes were conducted in R (v4.4.3) using the *pheatmap* and *stats* packages.

### 4.8 Construction of the Ecological Early Warning Index

To quantify behavioral perturbations in a unified manner, we developed a data‐driven EWI using a multi‐stage dimensionality reduction pipeline. Raw behavioral metrics were robustly processed using Winsorization (2% tails) and outlier filtering (>2× IQR), then Z‐score normalized relative to control samples, with metric directionality standardized such that positive values consistently indicated deviation from baseline. Metrics were organized into five ecological functional domains using a semi‐supervised, data‐assisted assignment procedure. Within each domain, dimensionality was reduced by selecting a compact set of non‐redundant metrics (3‐4 per domain) ranked by effect size (Hedges' g), followed by effect‐size–weighted aggregation to construct domain‐level indices. To identify the most informative functional dimensions while limiting overfitting, domain contributions were further pruned using L1‐regularized logistic regression. The final EWI was defined as the linear predictor of the refined model, rescaled to the range [0,1]. Discriminative performance was assessed using 5‐fold cross‐validation (area under the ROC curve), and anomaly thresholds were defined as 2 standard deviations above the control mean.

### 4.9 Polarization Parameter Extraction

The polarization accuracy and repeatability are calibrated using a rotating‐linear‐polarizer. The input linear polarization angle was varied from $0^{\circ }$ to $170^{\circ }$ in $10^{\circ }$ increments, with 10 repeated acquisitions targeted at each angle. The recovered AoP showed a bias of $-0.001^{\circ }$, MAE of $0.50^{\circ }$, RMSE of $0.66^{\circ }$, and 95% absolute error of $1.21^{\circ }$. For fully linearly polarized input, the calibrated DoLP was 1.000 on average, with an MAE of 0.015 from the ideal value of 1. The within‐angle repeatability was high, with an AoP SD of $0.025^{\circ }$ and a DoLP SD of $8.1\times 10^{-4}$ across repeated acquisitions, details are listed in Table S6.

To map structural anisotropy, raw polarization holograms were demodulated into Stokes parameters ($S_{0},S_{1},S_{2}$) from the four sub‐pixel channels. The angle of polarization (AoP) and degree of linear polarization (DoLP) were computed as:

(2) $$\begin{array}{cc} \mathrm{AoP} & =\frac{1}{2}\tan^{-1}\left(\frac{S_{2}}{S_{1}}\right), \end{array}$$

(3) $$\begin{array}{cc} \mathrm{DoLP} & =\frac{\sqrt{S_{1}^{2}+S_{2}^{2}}}{S_{0}}, \end{array}$$

where $S_{0}=I_{0^{\circ }}+I_{90^{\circ }}$, $S_{1}=I_{0^{\circ }}-I_{90^{\circ }}$, and $S_{2}=I_{45^{\circ }}-I_{135^{\circ }}$, with $I_{0^{\circ }},I_{45^{\circ }},I_{90^{\circ }},I_{135^{\circ }}$ denoting the four polarization subimages. These parameters serve as label‐free optical readouts of the spatial organization and strength of endogenous polarization contrast.

### 4.10 Calcium Imaging Validation

Intracellular calcium signaling was independently validated by laser scanning confocal microscopy (LSCM). Rotifers were loaded with the calcium‐sensitive dye Fluo‐4 AM (Thermo Fisher Scientific, USA) following standard protocols. The dye was prepared by dissolving 50μg in 50μL DMSO containing 1% Pluronic F‐127 (Molecular Probes, USA), sonicated for 15min, and applied via bath‐loading to reach a final concentration of 10 μM. Samples were imaged using an excitation wavelength of 488nm on an LSCM system. Fluorescence images were used to assess spatial patterns of calcium activity and to correlate functional perturbations assessed from label‐free PHIM measurements. Relative fluorescence changes were calculated as $\Delta F/F_{0}$ to quantify cytosolic calcium transients.

### 4.11 Statistical Analysis

Data are presented as mean ± standard deviation (s.d.) or as box plots showing the median and interquartile range. For comparisons among more than two groups, one‐way ANOVA followed by Tukey's multiple‐comparison test was used. Statistical significance was defined as p<0.05. Significance levels are denoted as follows: ns, not significant; *p<0.05; ****p<0.01; ***p<0.001; and ****p<0.0001.

All statistical analyzes and plots were generated using GraphPad Prism 10 and custom MATLAB/Python scripts. Exact sample sizes, biological replicates, statistical units, and inclusion criteria for each quantitative analysis are reported in Table S4.

## Author Contributions

Conceptualization: Y. L. and E. Y. L.; methodology: Y. L., Y. Z., and Y. H.; investigation: Y. L., Y. Z., J. C., R. C., C. W.; visualization: Y. L. and J. C.; writing (original draft): Y. L.; writing (review and editing): Y. L., Y. Z., J. C., R. C., C. W., Y. H., E. Y. L.; resources: J. K. F., K. T., and E. Y. L.; supervision and funding acquisition: E. Y. L. All authors reviewed and approved the manuscript.

## Conflicts of Interest

The authors declare no conflicts of interest.

## Data and Materials Availability

The datasets used and/or analyzed during the current study are available at https://github.com/yuxingli‐HKU/PHIM.

## Supporting information


**Supporting File**: advs77832‐sup‐0001‐SuppMat.pdf.
