# Supplementary material for: Polarization Holographic 4D Phenotyping Reveals Nanoplastic Induced Dysfunction in Zooplankton
Source: Adv Sci (Weinh). 2026 Oct 4:e77832. Online ahead of print. doi: 10.1002/advs.77832 (PMC13635408; doi:10.1002/advs.77832)
Supplement: Supplementary file 1 [file ADVS-9999-e77832-s001.pdf]

Supplementary Information: Polarization holographic 4D phenotyping reveals nanoplastic-induced dysfunction in zooplankton

Yuxing Li[1*], Yanmin Zhu[1], Jingyan Chen[1], Rongzhou Chen[1],
Yuen-Wa Ho[2], Chutian Wang[1], James Kar-Hei Fang[2,3,5], Kevin Tsia[1,4],
Edmund Y. Lam[1*]

[1]Department of Electrical and Electronic Engineering, The University of Hong Kong, Pokfulam, Hong Kong, China

[2]Department of Food Science and Nutrition, The Hong Kong Polytechnic University, Hung Hom, Hong Kong, China

[3]State Key Laboratory of Marine Environmental Health, City University of Hong Kong, Kowloon Tong, Hong Kong, China

[4]Advanced Biomedical Instrumentation Centre, Hong Kong Science Park, Shatin, New Territories, Hong Kong, China

[5]PolyU-BGI Joint Research Centre for Genomics and Synthetic Biology in Global Ocean Resources, The Hong Kong Polytechnic University, Hung Hom, Hong Kong, China

[*]Corresponding author. Email: yuxingli@hku.hk, elam@eee.hku.hk

Contents

Supplementary notes 1 and 2

Supplementary figures 1 to 18

Supplementary tables 1 to 8

Supplementary Note 1: Adaptive multimetric holographic autofocusing (AMHA)

To robustly determine the true object plane for each region of interest (ROI) without manual tuning, we developed an AMHA algorithm that synergistically integrates physical back-propagation using the angular-spectrum method with dynamic, data-driven focus metric weighting. Each recorded hologram $U(x, y; 0)$ is numerically propagated across the axial range $[z_{\min}, z_{\max}]$ at intervals of Δz, using the Fourier-domain Fresnel diffraction integral (*1*):

$$U(x, y; z) = \mathcal{F}^{-1}\Big\{\mathcal{F}\{U(x, y; 0)\} \exp\Big[i2\pi z\sqrt{\lambda^{-2} - f_x^2 - f_y^2}\Big]\Big\}, \tag{S1}$$

where $\mathcal{F}$ denotes the 2D Fourier transform, λ the illumination wavelength, and (f_x, f_y) represents spatial frequency variables. We used micrometer-level step sizes to ensure adequate depth coverage.

For each propagated slice $U(x, y; z)$, we compute its amplitude $A_z(x, y) = |U(x, y; z)|$, followed by high-pass filtering via morphological opening (disk radius 5 px) and contrast stretching, yielding an enhanced image $I_{\text{hp}}(x, y)$ that accentuates fine structural details. While the focus metrics employed are derived from high-pass filtering principles, each captures distinct aspects of image sharpness. Specifically, Tenengrad score $F_T(z)$ reflects local directional edge strength using the squared Sobel gradient magnitude (*2*); Laplacian variance $F_L(z)$ captures fine-scale intensity fluctuations through second-order spatial derivatives (*3*); Brenner function $F_B(z)$ efficiently responds to broader grayscale transitions, offering noise-robust contrast estimation (*4*).

Three complementary focus metrics are then evaluated on I_{hp}:

$$F_T(z) = \frac{1}{N}\sum_{i,j}\left[\nabla_{\text{Sobel}} I_{\text{hp}}(i, j)\right]^2, \tag{S2}$$

$$F_L(z) = \frac{1}{N}\sum_{i,j}\left[\Delta I_{\text{hp}}(i, j) - \mu_{\Delta I}\right]^2, \tag{S3}$$

$$F_B(z) = \frac{1}{N}\sum_{i,j}\left[I_{\text{hp}}(i, j) - I_{\text{hp}}(i+2, j)\right]^2, \tag{S4}$$

where $\nabla_{\text{Sobel}} I_{\text{hp}}(i, j) = \sqrt{\left(\nabla_x I_{\text{hp}}(i, j)\right)^2 + \left(\nabla_y I_{\text{hp}}(i, j)\right)^2}$ is the gradient magnitude computed via the Sobel operator in both x and y directions, Δ is the discrete Laplacian operator, N is the total number of pixels in the image, and $\mu_{\Delta I}$ is the mean value of ΔI_{hp}. Each metric is normalized as

$\tilde{F}_k(z) = \frac{F_k(z) - \min_z F_k(z)}{\max_z F_k(z) - \min_z F_k(z) + \epsilon}$, with ϵ a small constant (e.g., 10^{-8}) to ensure numerical stability. The dynamic range $R_k = \max_z F_k(z) - \min_z F_k(z)$ defines the adaptive weight $w_k = \frac{R_k}{\sum_{m \in \{T,L,B\}} R_m}$, which balances the relative sensitivity of each metric. The final composite focus score is given by $F_C(z) = \sum_{k \in \{T,L,B\}} w_k \tilde{F}_k(z)$.

The optimal focal plane is identified as $z^* = \arg\max_z F_C(z)$, and a final angular-spectrum reconstruction at z^* yields the sharply focused amplitude image $A_{z^*}(x, y)$, enabling high-fidelity downstream analyses of trajectory and morphology.

Supplementary Note 2: Definition of 27 locomotion metrics

Let a trajectory be a sequence of N positions $\mathbf{r}_i = (x_i, y_i, z_i)$ $(i = 1, \dots, N)$ sampled at a constant interval Δt. Define the step vector

$$\Delta\mathbf{r}_i = \mathbf{r}_{i+1} - \mathbf{r}_i, \quad \Delta s_i = \|\Delta\mathbf{r}_i\|_2, \quad i = 1, \dots, N-1, \tag{S5}$$

the instantaneous speed

$$v_i = \frac{\Delta s_i}{\Delta t}, \quad i = 1, \dots, N-1, \tag{S6}$$

and the instantaneous acceleration

$$a_i = \frac{v_{i+1} - v_i}{\Delta t}, \quad i = 1, \dots, N-2. \tag{S7}$$

The 3-D turning angle between successive steps is

$$\theta_i = \cos^{-1}\Big(\frac{\Delta\mathbf{r}_i \cdot \Delta\mathbf{r}_{i+1}}{\|\Delta\mathbf{r}_i\| \, \|\Delta\mathbf{r}_{i+1}\| + \varepsilon}\Big), \tag{S8}$$

with $\varepsilon \ll 1$ to avoid division by zero. Planar headings are

$$\phi_i = \mathrm{atan2}(\Delta y_i, \Delta x_i), \quad i = 1, \dots, N-1.$$

S1.1 Translational kinematics

(P1: TK1) Total path length L

$$L = \sum_{i=1}^{N-1} \Delta s_i.$$

(P2: TK2) Net displacement D

$$D = \|\mathbf{r}_N - \mathbf{r}_1\|_2.$$

(P3: TK3) Directionality index DI

$$\mathrm{DI} = \frac{D}{L} \in [0, 1].$$

(P4: TK4) Average speed $\bar{v}$

$$\bar{v} = \frac{1}{N-1} \sum_{i=1}^{N-1} v_i.$$

S1.2 Acceleration statistics

(P5: AS1) Average acceleration $\bar{a}$

$$\bar{a} = \frac{1}{N-2} \sum_{i=1}^{N-2} a_i.$$

(P6: AS2) Acceleration variance $\mathrm{Var}(a_i)$

$$\mathrm{Var}(a_i) = \frac{1}{N-2} \sum_{i=1}^{N-2} (a_i - \bar{a})^2.$$

(P7: AS3) Acceleration stability AccelStability

$$\mathrm{AccelStability} = \frac{\sqrt{\mathrm{Var}(a_i)}}{\bar{a} + \varepsilon}.$$

(P8: AS4) Maximum acceleration $a_{\max}$

$$a_{\max} = \max_{1 \le i \le N-2} |a_i|.$$

S1.3 Rotation and turning

(P9: RT1) Average angular speed $\bar{\omega}$

$$\bar{\omega} = \frac{1}{N-2} \sum_{i=1}^{N-2} \frac{\theta_i}{\Delta t}.$$

(P10: RT2) Average curvature $\bar{\kappa}$ Define $\kappa_i = \theta_i / (\Delta s_{i+1} + \varepsilon)$. Then

$$\bar{\kappa} = \frac{1}{N-2} \sum_{i=1}^{N-2} \kappa_i.$$

(P11: RT3) Turn angle variance Var_{xy} In the xy-plane, $\mathrm{Var}_{xy} = \mathrm{Var}(\mathrm{wrapToPi}(\phi_{i+1} - \phi_i))$.

(P12: RT4) Circular variance CV Let $R = \left|\langle e^{i\phi_i} \rangle\right|$. Then

$$\mathrm{CV} = 1 - R.$$

(P13: RT5) High-turn ratio HTR

$$\mathrm{HTR} = \frac{\#\{\theta_i > \theta_{\mathrm{th}}\}}{N-2}, \quad \theta_{\mathrm{th}} = \pi/6.$$

(P14: RT6) Meandering index MI

$$\mathrm{MI} = \frac{\sum_{i=1}^{N-2} \theta_i}{L} \quad [\mathrm{rad} \cdot \mathrm{length}^{-1}].$$

S1.4 Path complexity and diffusion

(P15: PC1) 3D tortuosity $\mathcal{T}_{3D}$

$$\mathcal{T}_{3D} = \frac{L}{D}, \quad (\mathcal{T}_{3D} = 0 \text{ if } D = 0).$$

(P16: PC2) xy-plane tortuosity $\mathcal{T}_{xy}$ $\quad \mathcal{T}_{xy} = L_{xy}/D_{xy}$

$$\text{with } L_{xy} = \sum \sqrt{\Delta x_i^2 + \Delta y_i^2},\ D_{xy} = \sqrt{(x_N - x_1)^2 + (y_N - y_1)^2}.$$

(P17: PC3) Path volume 3D Convex hull volume of $\{\mathbf{r}_i\}$.

(P18: PC4) Effective diffusion coefficient D_{eff} From MSD linear fit: $D_{\mathrm{eff}} = \frac{\mathrm{slope}}{2n}$.

(P19: PC5) Diffusion exponent α Slope of $\log \mathrm{MSD}$ vs. $\log \tau$.

S1.5 Velocity fluctuation and spectra

(P20: VS1) Speed variance $\mathrm{Var}(v_i)$

(P21: VS2) Speed stability SpeedStability $\frac{\sqrt{\mathrm{Var}(v_i)}}{\bar{v}+\varepsilon}$.

(P22: VS3) Speed entropy $-\sum_{j=1}^{B} p_j \ln p_j \quad (B = 10)$.

(P23: VS4) Peak frequency Dominant non-zero Fourier frequency of $v_i - \bar{v}$.

S1.6 Behavioral state distribution

(P24: BS1) Freeze time ratio $\frac{\#\{v_i < v_{\mathrm{freeze}}\}}{N-1}$.

(P25: BS2) Swim time ratio $\frac{\#\{v_{\mathrm{freeze}} \le v_i < v_{\mathrm{rapid}}\}}{N-1}$.

(P26: BS3) Rapid time ratio $\frac{\#\{v_i \geq v_{\text{rapid}}\}}{N-1}$.

(P27: BS4) Freeze event count Number of contiguous segments with $v_i < v_{\text{freeze}}$.

Symbol definitions

Δt: sampling interval; ε: small constant; wrapToPi: maps angles to $(-\pi, \pi]$.

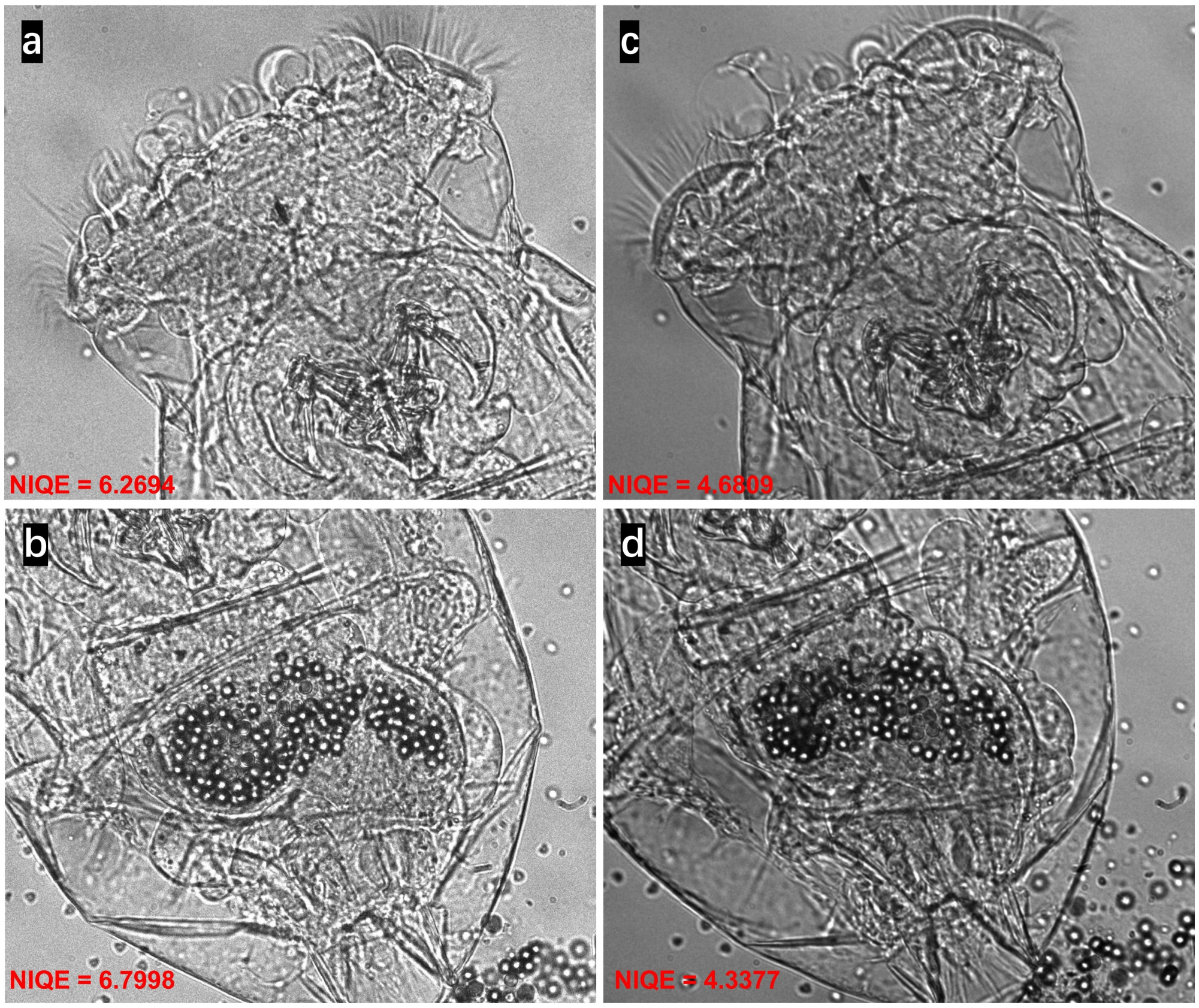

Figure S1: **Polarization-based denoising of holographic images.** The intensity distribution of (a) corona and (b) body images exhibiting reflection and scattering noise. (c) and (d) show the same regions after polarization optimization. Comparison of naturalness image quality evaluator (NIQE) scores before and after denoising, demonstrating a marked improvement in perceived image quality (lower NIQE denotes better quality).

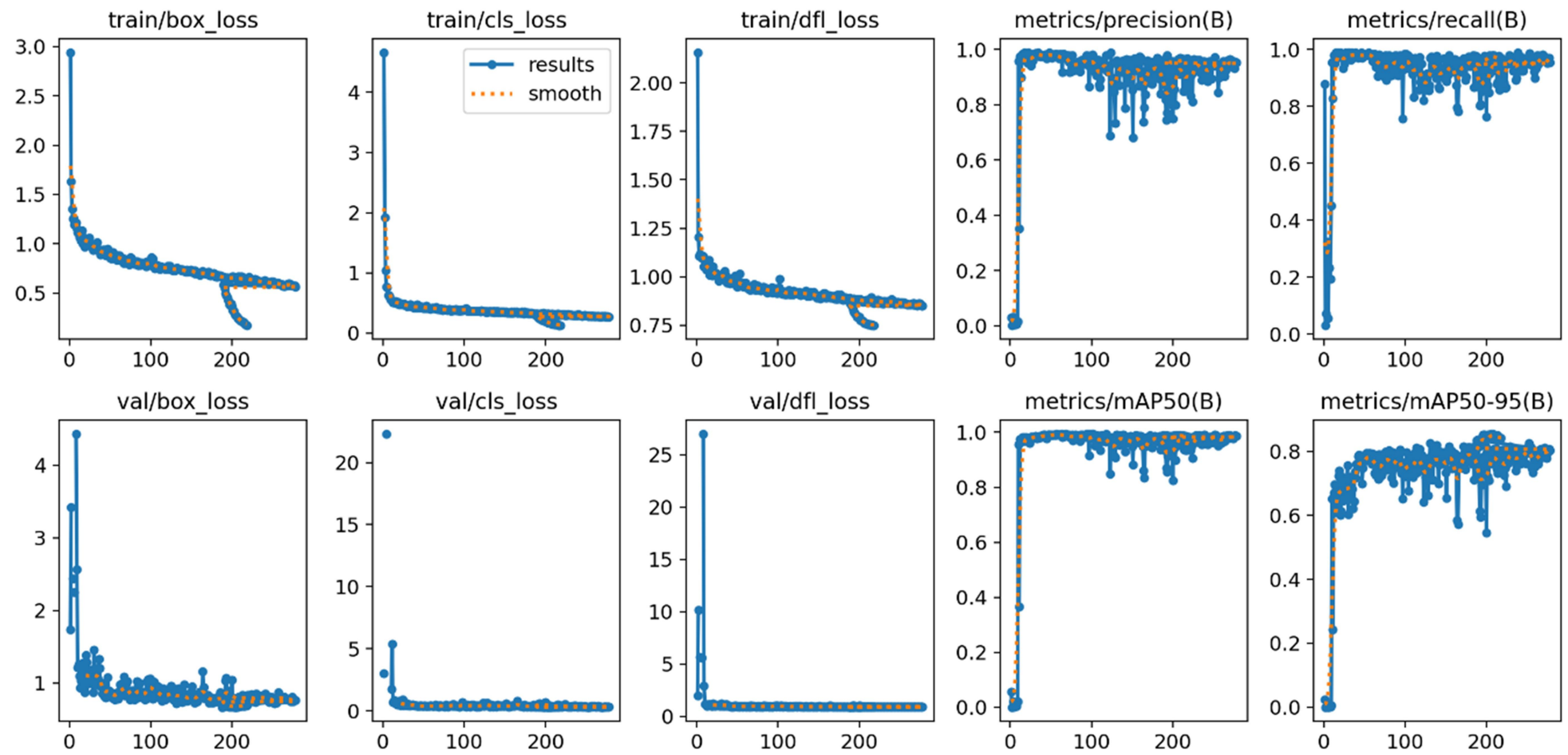

Figure S2: **Training performance of the deep-learning ROI detector.** The evolution of training and validation loss curves over 230 epochs, and corresponding precision and recall metrics, demonstrating convergence and high detection accuracy for automatic ROI localization.

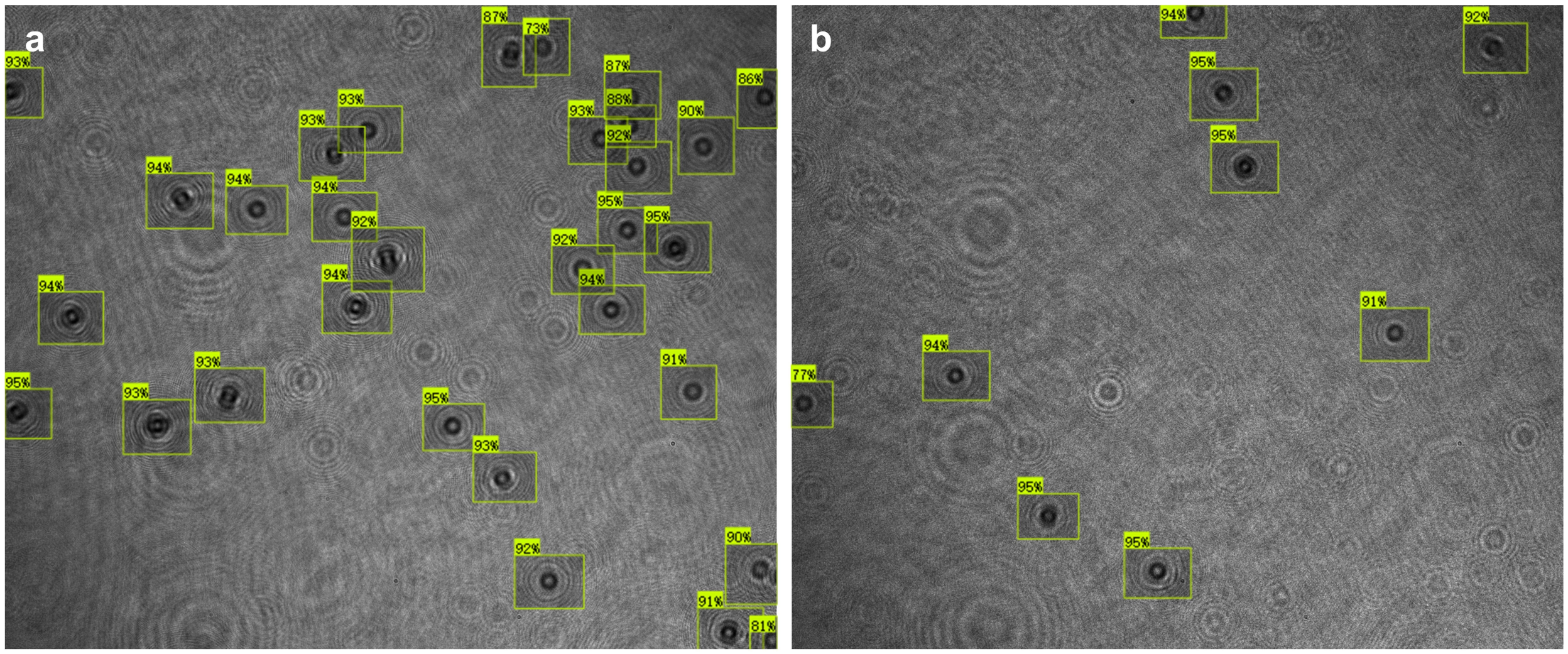

Figure S3: **ROI detection robustness under different rotifer densities.** (a) High-density field containing 31 rotifers with multiple instances of spatial overlap. (b) Low-density field containing 9 rotifers without overlap. Detected bounding boxes and confidence scores demonstrate that the lightweight YOLO11n detector can robustly identify individual rotifers across density conditions.

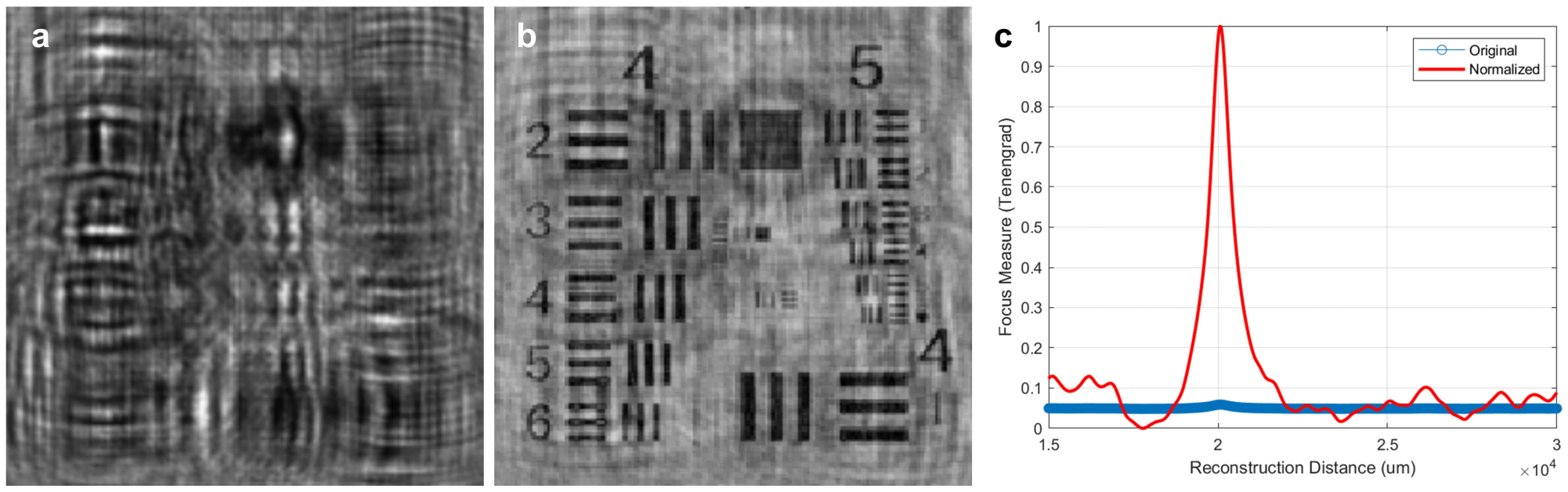

Figure S4: **USAF 1951 resolution target reconstruction and focus metric assessment.** (a) Raw inline hologram of the target. (b) Back-propagated amplitude image after digital reconstruction. (c) Tenengrad focus metric plotted across propagation distances, with a clear peak indicating optimal focus for this thin target.

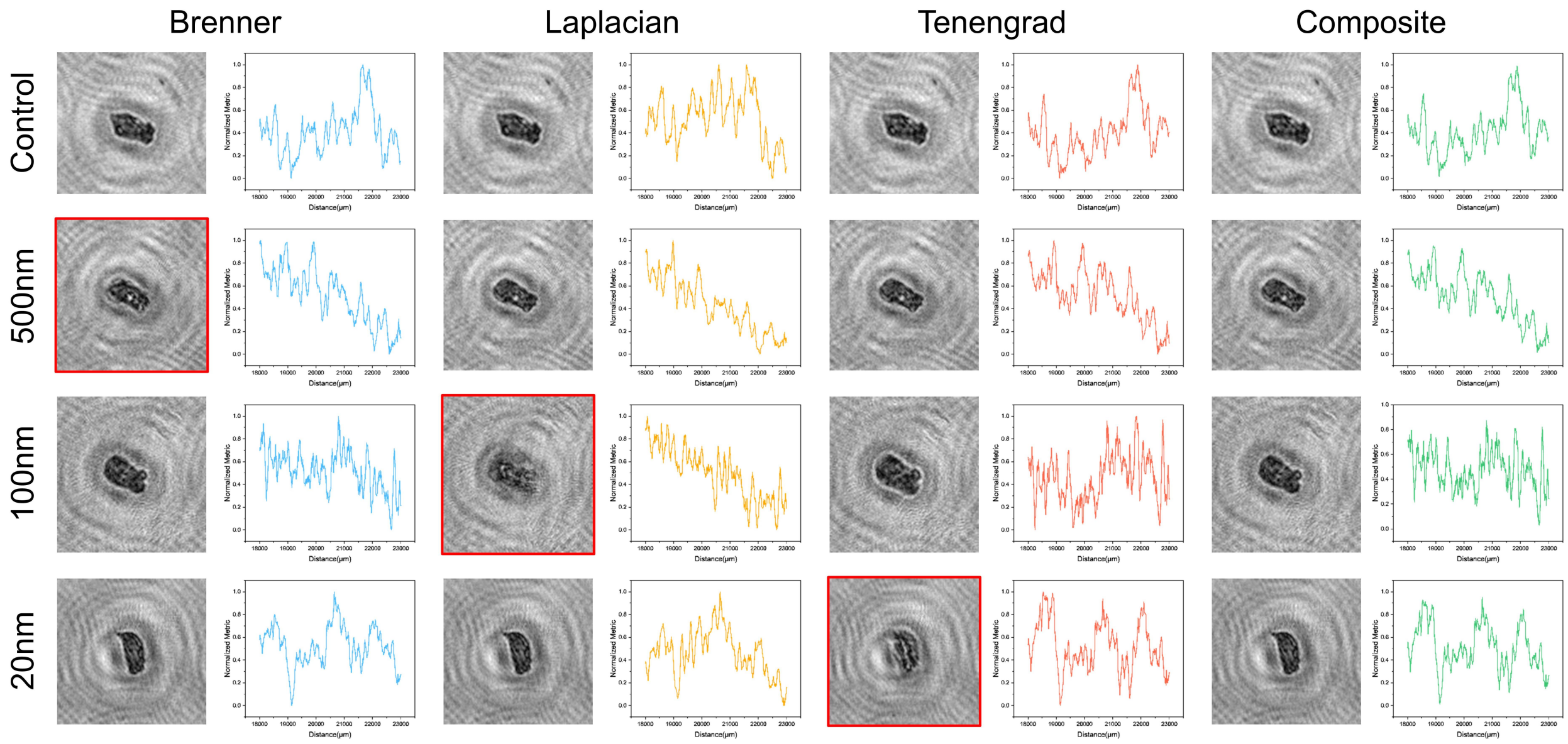

Figure S5: **Comparison of autofocusing metrics under nanoparticle exposure.** Each row shows a different treatment condition, control, 500 nm, 100 nm and 20 nm polystyrene (PS) beads. Columns correspond to three common sharpness measures, Brenner, Laplacian, Tenengrad, and a composite metric. For each metric, the left subpanel is the image crop used for focus evaluation; the right subpanel plots the normalized metric value (distance in µm). Red-outlined crops indicate cases where that single metric fails to peak at the true focus position. In contrast, the composite metric (rightmost column) consistently identifies the correct focus across all treatments.

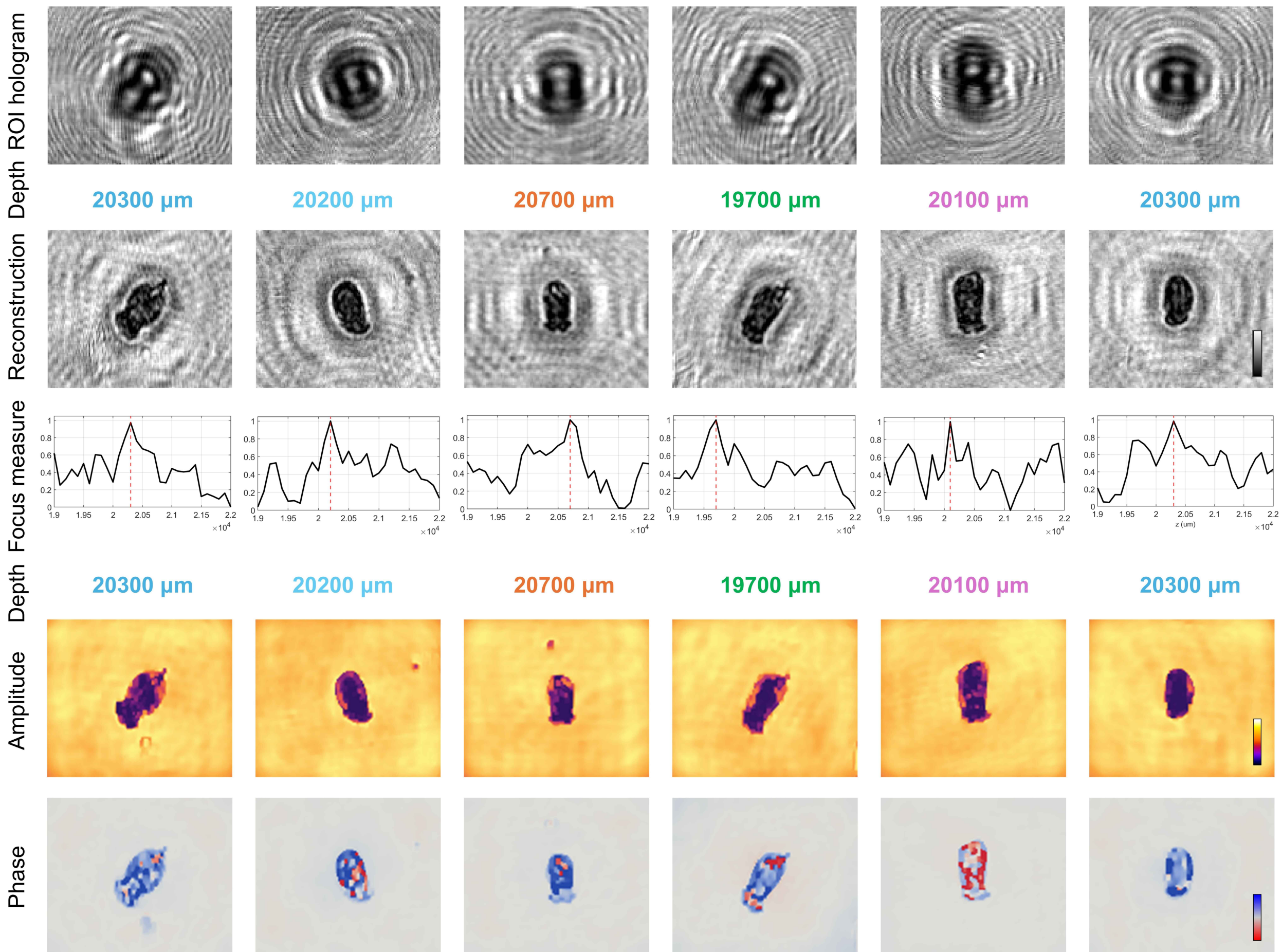

Figure S6: **Twin-image suppression of AMHA-selected ROIs.** Representative rotifer ROI holograms were first digitally refocused by AMHA, and the best-focus planes were identified from the focus-measure curves. The corresponding ROI holograms were then processed using the constrained complex total variation (CCTV) phase-retrieval algorithm (*5*) to suppress the in-line holographic twin-image background. The CCTV-recovered amplitude and phase images show compact rotifer structures with reduced defocused fringe background, supporting that the residual twin-image contribution did not dominate the focus detection or reconstructed morphology.

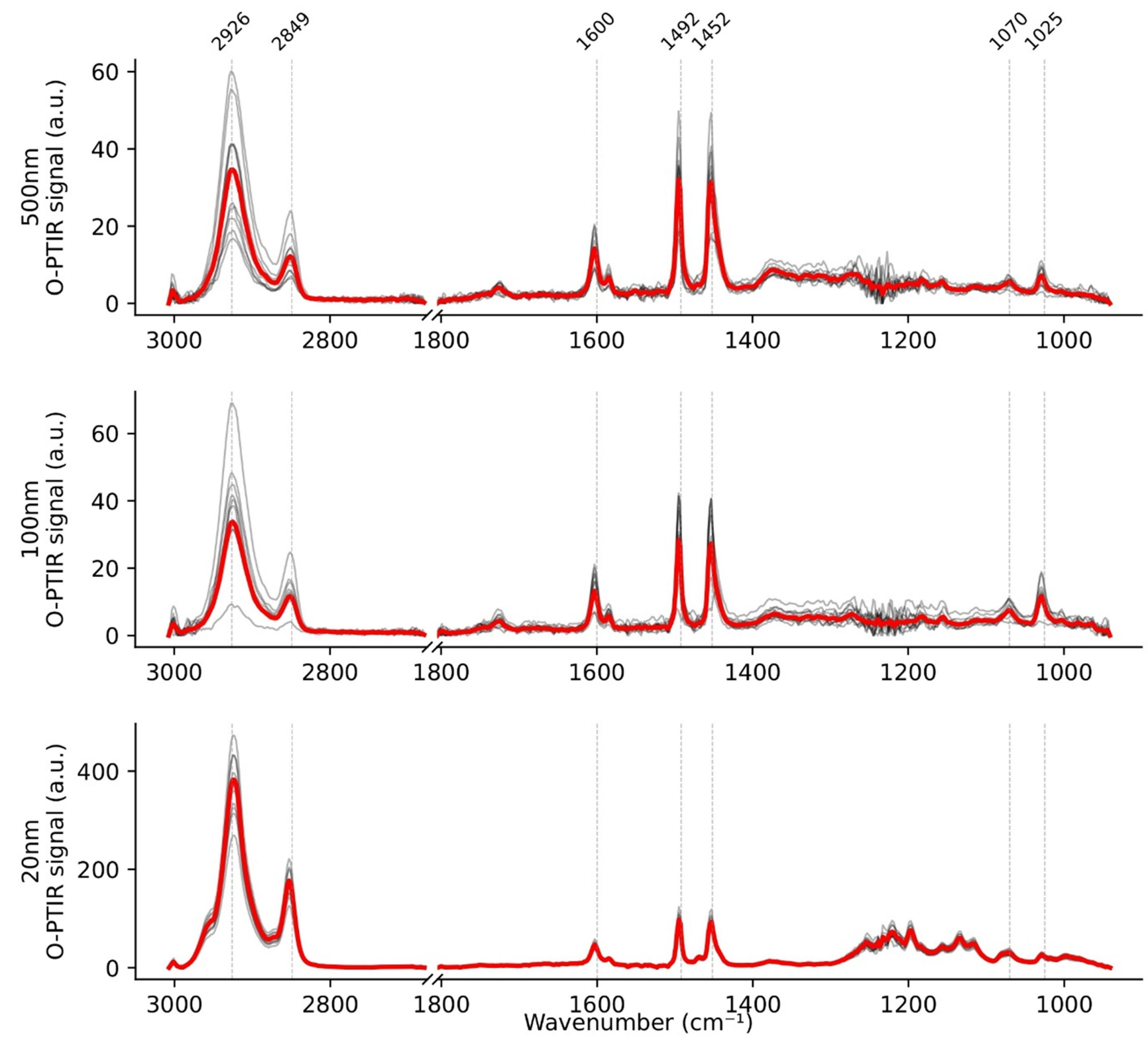

Figure S7: **Optical photothermal infrared (O-PTIR) spectra.** PS nanoplastics for the three size classes (500 nm, 100 nm and 20 nm). Characteristic PS absorption bands are preserved across all particle sizes, confirming chemical integrity after size reduction.

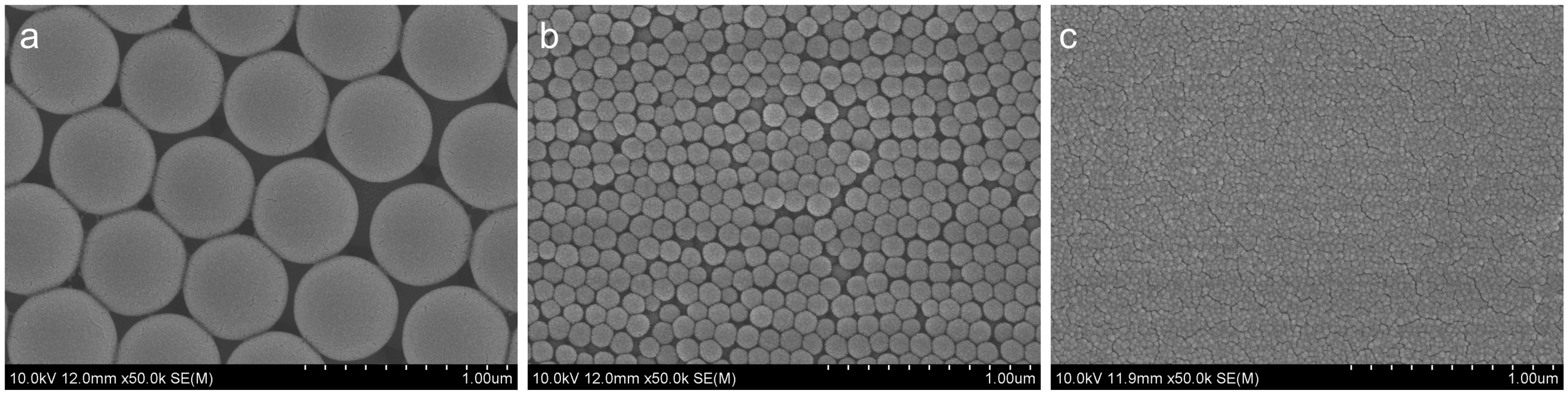

Figure S8: **Representative scanning electron microscopy (SEM) images.** PS nanoplastics with nominal diameters of 500 nm (a), 100 nm (b) and 20 nm (c). All particles exhibit a uniform, spherical morphology and a narrow size distribution. Scale bars are indicated in each panel.

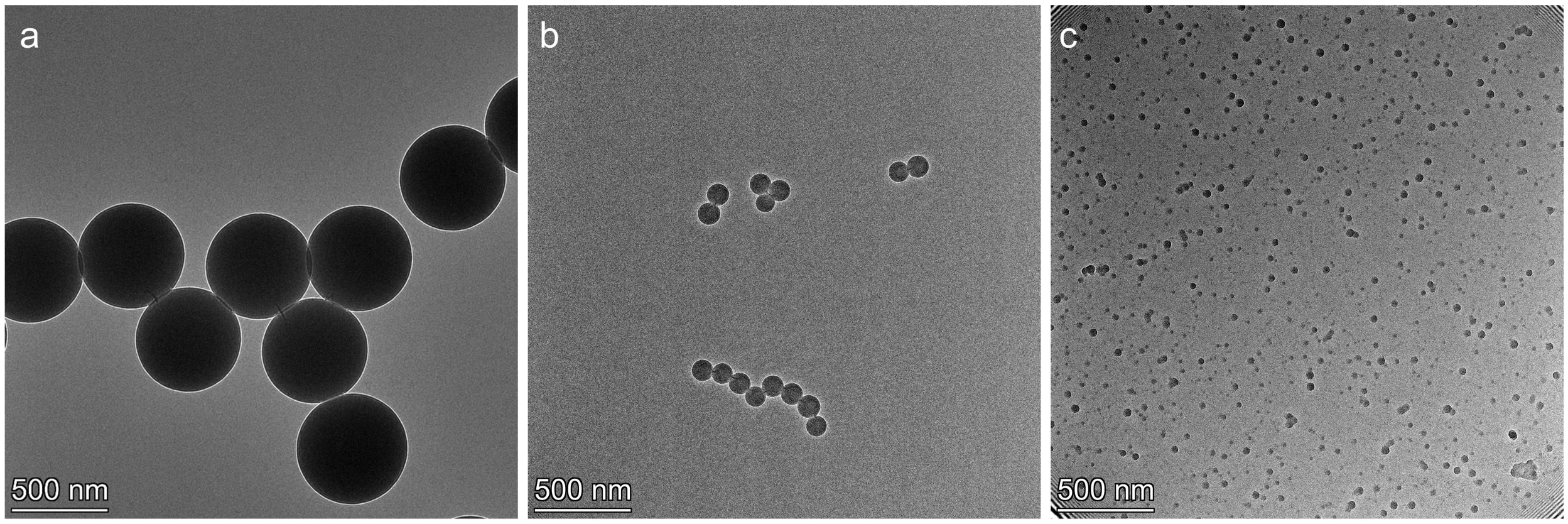

Figure S9: **Representative transmission electron microscopy (TEM) images.** PS particles at nominal sizes of 500 nm (a), 100 nm (b) and 20 nm (c). The 20 nm sample shows a relatively broader size distribution (16 nm–40 nm). Scale bars are shown in each panel.

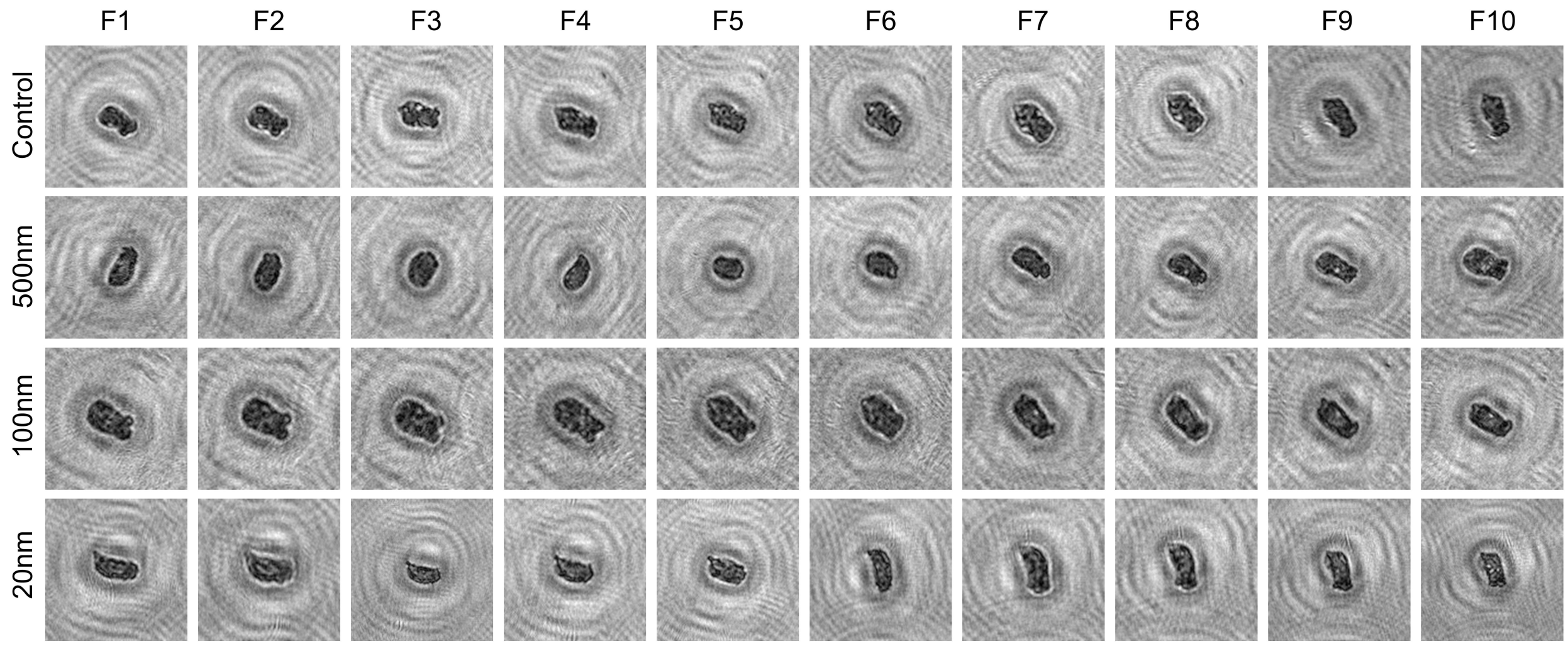

Figure S10: **Focus-stack frames F1–F10 for each nanoparticle treatment.** Rows correspond to control, 500 nm, 100 nm and 20 nm PS exposures; columns show ten sequential focal planes (F1–F10) reconstructed from the raw holograms. These crops illustrate the robust performance for composite autofocus metric evaluation.

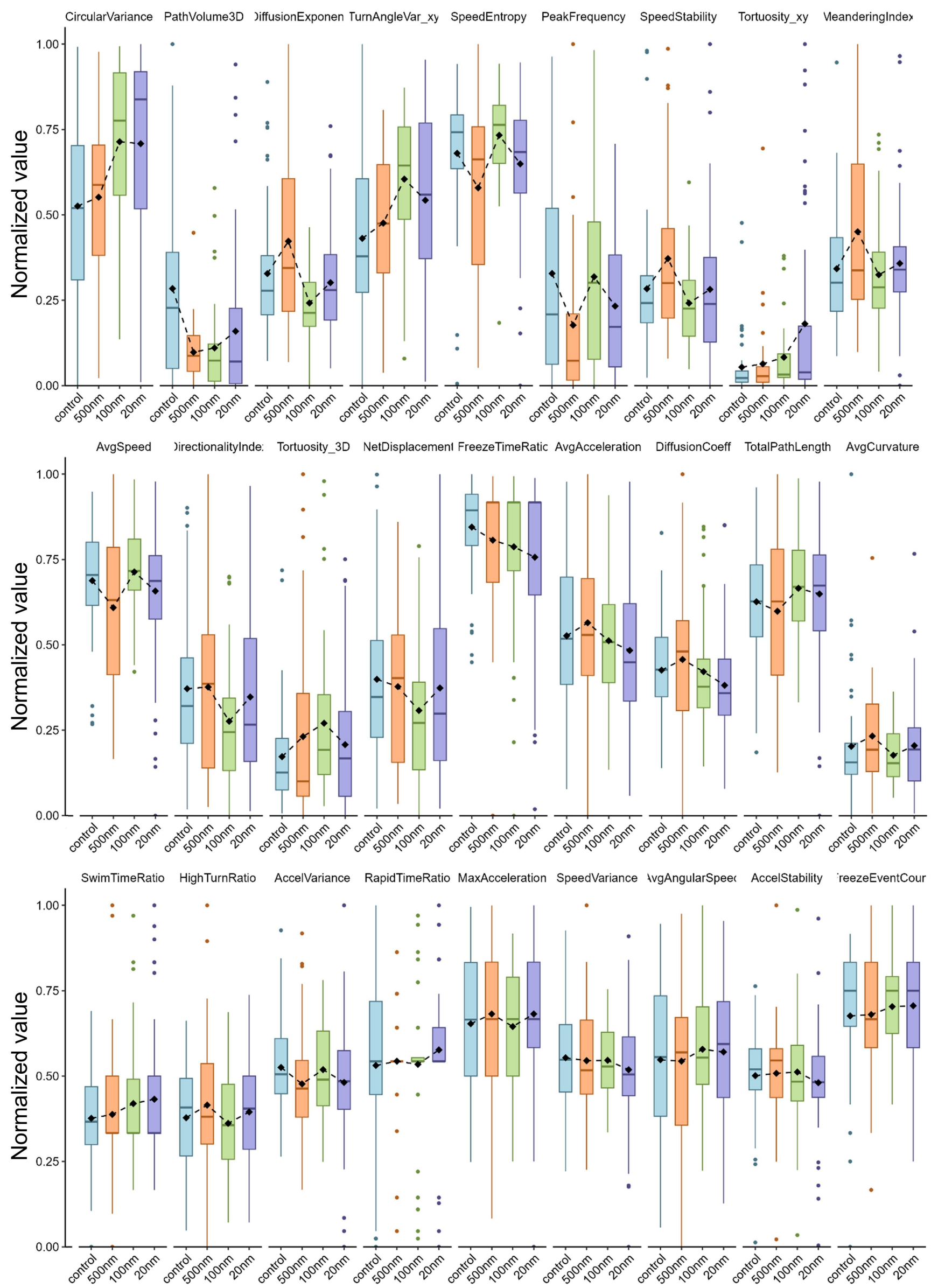

Figure S11: **Quantitative multivariate locomotion profiling across nanoparticle treatments.** Box-and-whisker plots of tracked rotifers per group (control, 500 nm, 100 nm, 20 nm beads; colors blue, orange, green, purple) showing normalized values of 27 trajectory features (arranged left-to-right, top-to-bottom). Changes in median and mean values reveal size-dependent alterations in swimming behavior.

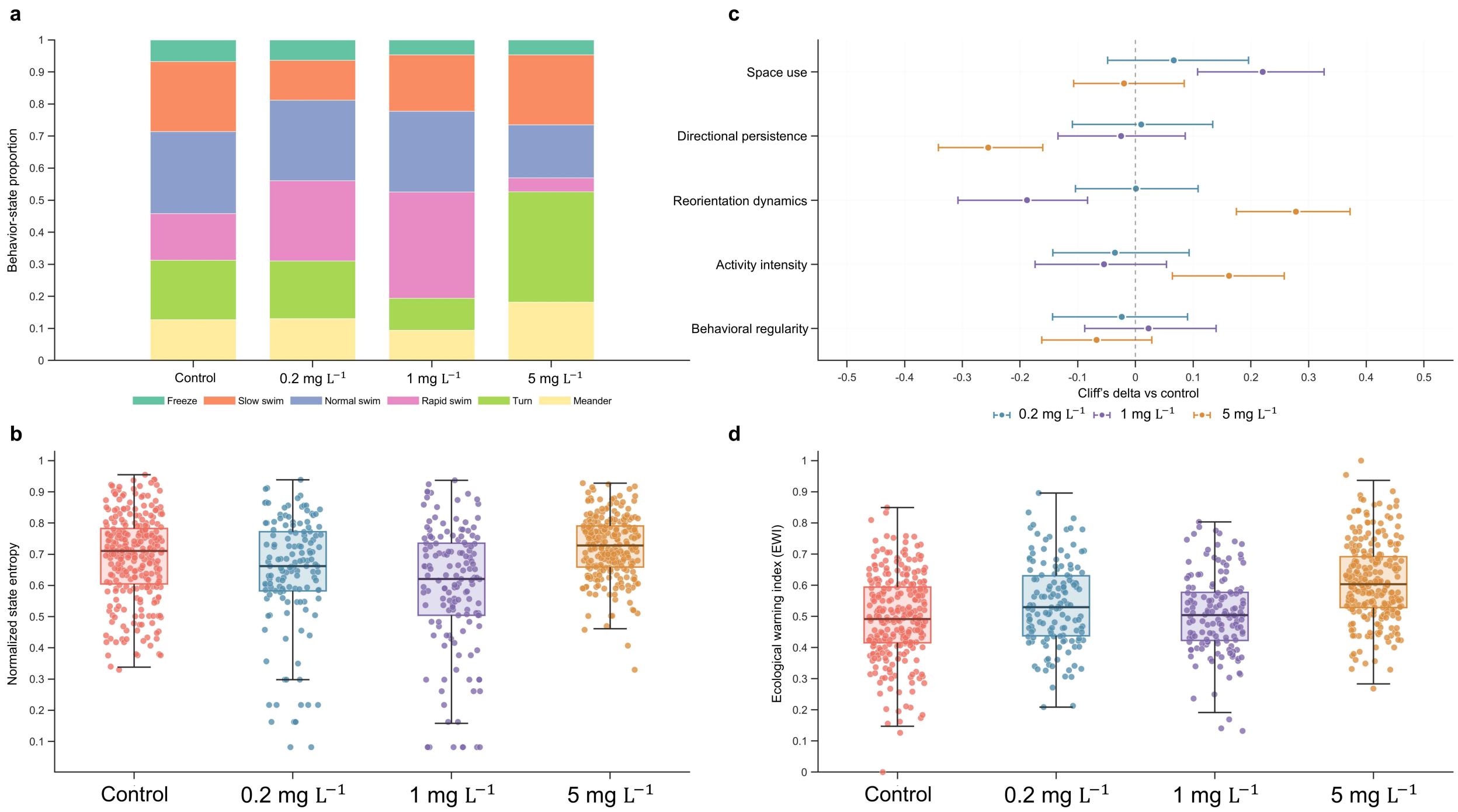

Figure S12: **Concentration-sensitivity validation for 20 nm PS nanoplastics.** Behavioral phenotypes of rotifers exposed to 20 nm PS nanoplastics at 0.2, 1, and 5 mg L^{-1}. (a) Behavioral-state occupancy across concentrations. (b) Normalized behavioral-state entropy. (c) Domain-level effect sizes relative to control, shown as Cliff's delta with confidence intervals. (d) Ecological warning index (EWI) across concentrations. Box plots show median and interquartile range.

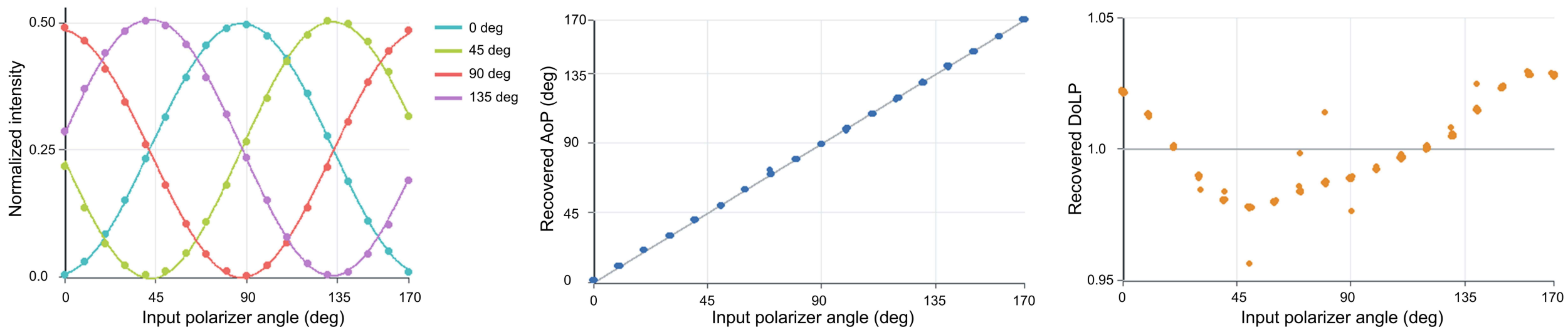

Figure S13: **Polarization calibration using a rotating linear polarizer.** A linear polarizer was rotated from 0° to 170° in 10° increments, and the four micro-polarizer channels were recorded at each angle. The four channel intensities followed the expected Malus-law modulation, the recovered AoP showed a linear response to the input polarizer angle, and the recovered AoP closely follows the imposed input angle, and recovered DoLP remains near unity for linearly polarized input, validating polarimetric accuracy and repeatability.

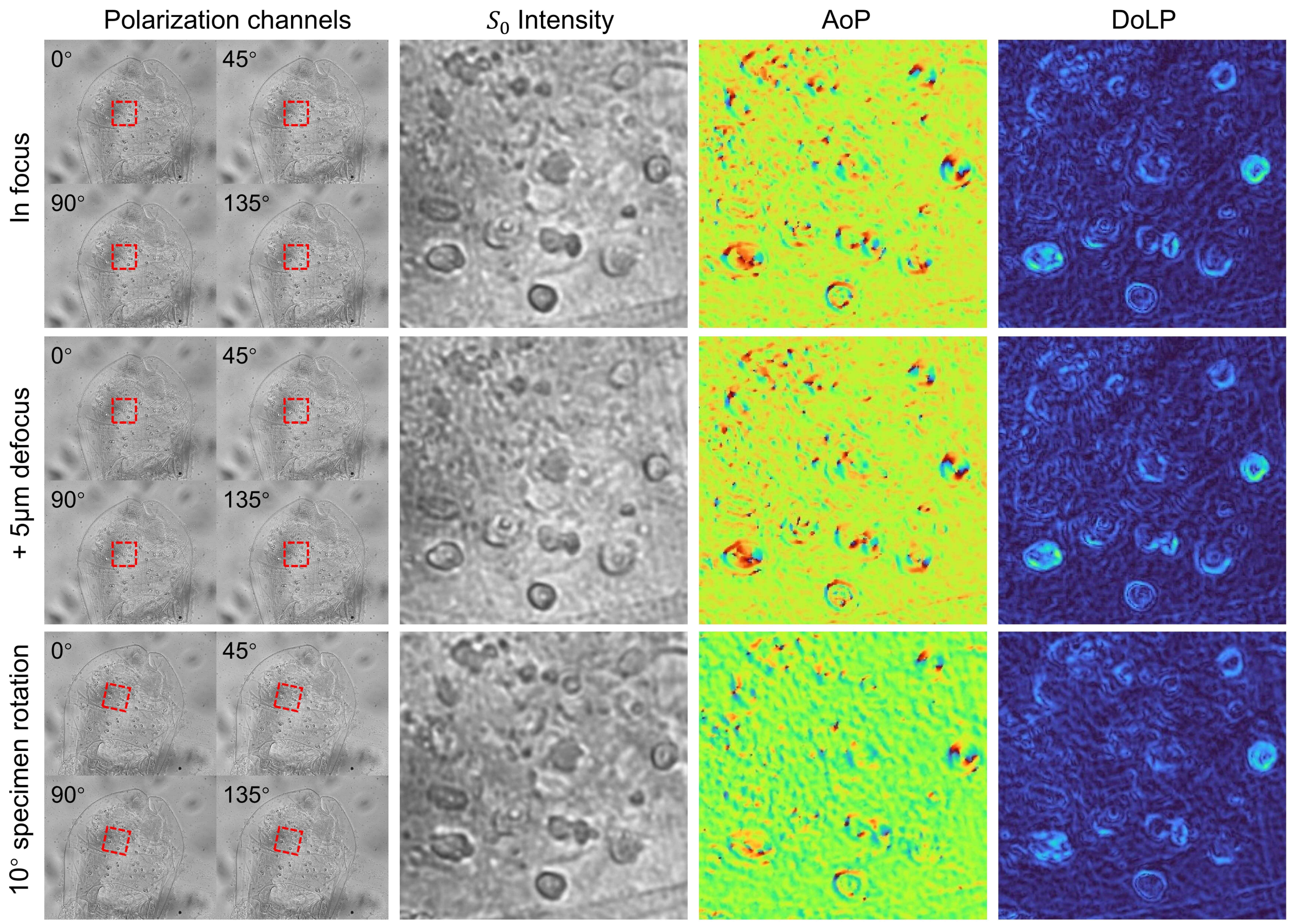

Figure S14: **Robustness of polarization maps under defocus and specimen rotation.** Four polarization-channel images were used to reconstruct S_0, AoP, and DoLP maps under in-focus, +5 μm defocus, and 10° rotation conditions. Vesicle-associated AoP/DoLP contrast was preserved across these perturbations, supporting that the observed polarimetric contrast is robust to small focal-plane and orientation variations.

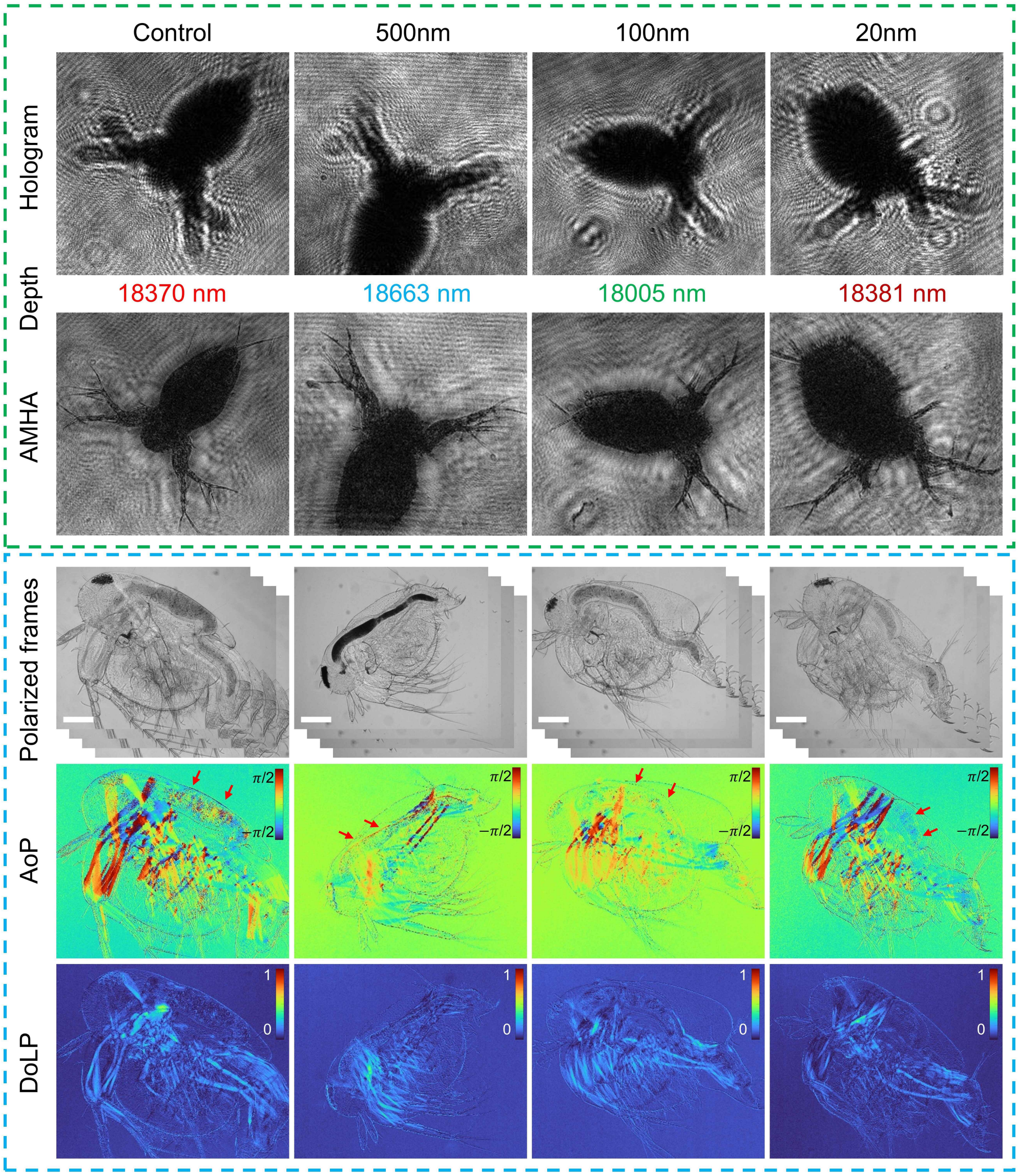

Figure S15: **Polarization holographic profiling of freshwater *Daphnia* under nanoplastic exposure.** Representative holograms, AMHA reconstructions, polarization-resolved frames, and derived AoP and DoLP maps of *Daphnia* in control and after exposure to 500 nm, 100 nm, and 20 nm PS nanoplastics. Refocusing depths are indicated. Red arrows in AoP maps highlight polarization anisotropy alterations along digestive and appendage structures. DoLP maps visualize particle-size-dependent perturbations of internal anisotropy.

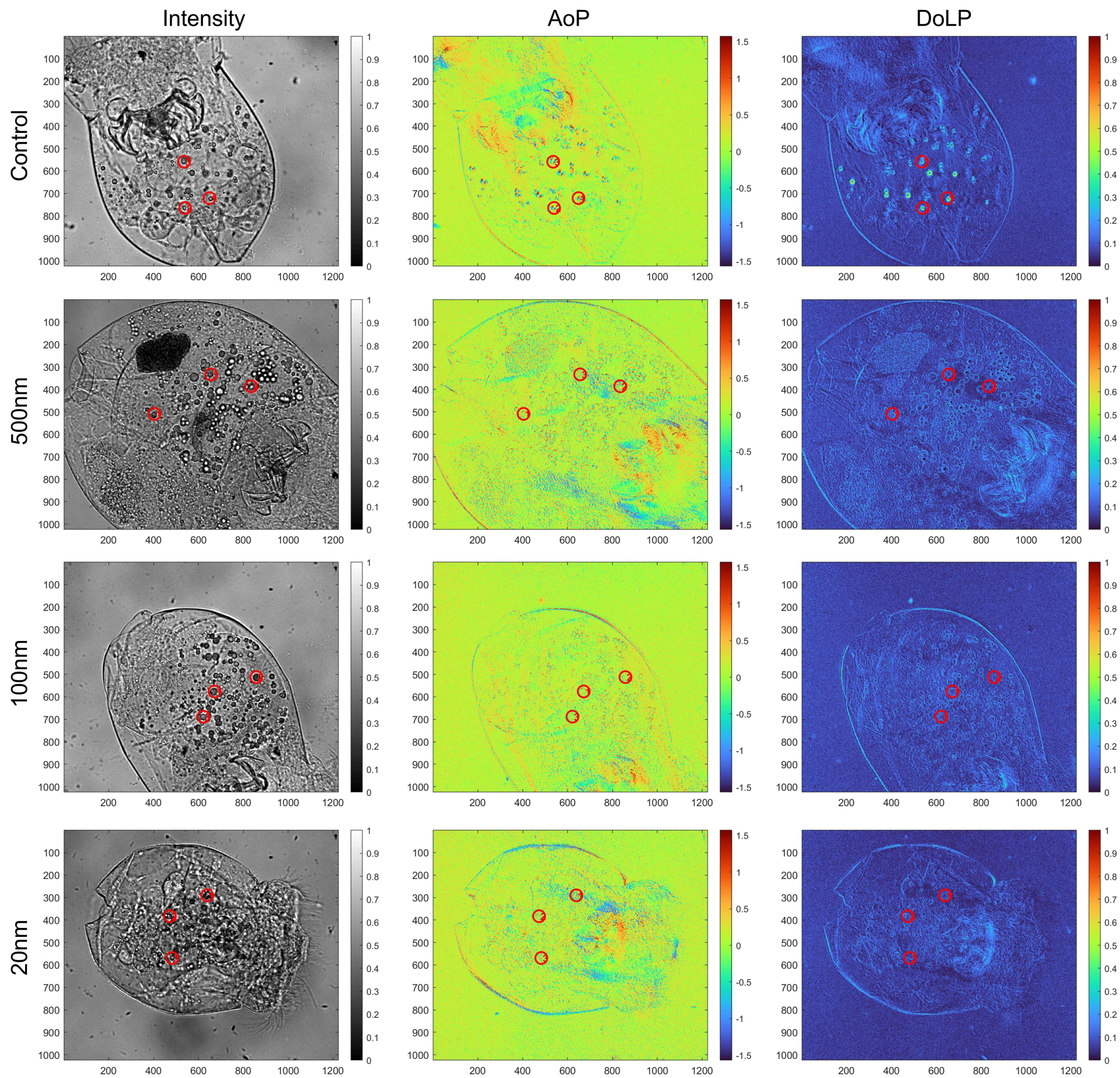

Figure S16: **Vesicle ROI selection for polarization quantification.** Representative S_0 intensity, AoP, and DoLP maps of control and nanoplastic-exposed rotifers. Red circles indicate digestive vesicle ROIs used for polarization quantification across the control, 500 nm, 100 nm, and 20 nm groups.

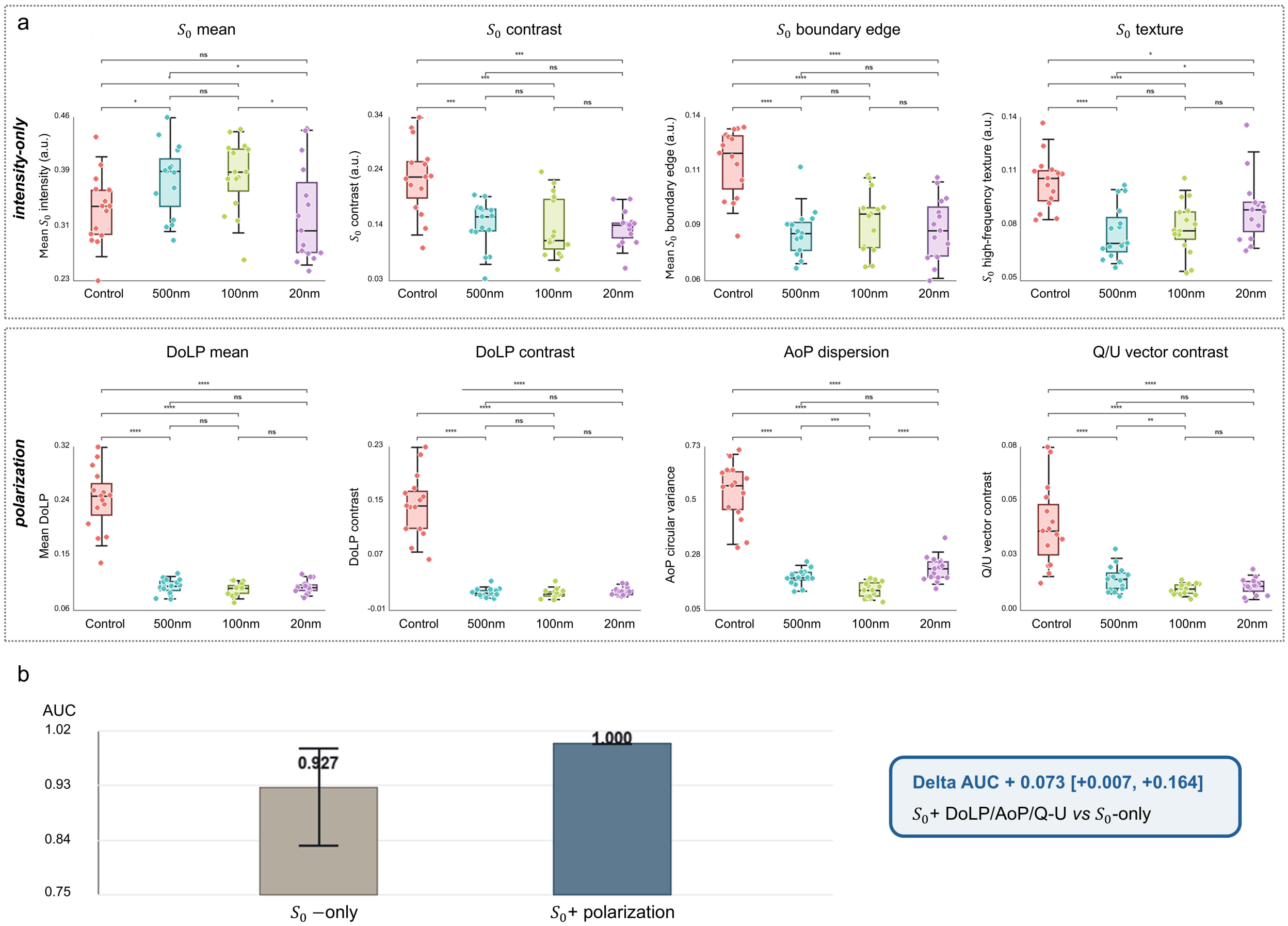

Figure S17: **Vesicle-level feature distributions and polarization feature ablation.** (a) Complete rotifer-level distributions of intensity-derived (S_0) and polarization-derived vesicle descriptors across exposure groups. Each point represents one rotifer-level mean from shared digestive-vesicle ROIs; pairwise significance was assessed by one-way ANOVA. (b) Repeat-aware feature-ablation analysis showing that adding DoLP, AoP, and Q/U-vector descriptors to the S_0 baseline increased the out-of-fold AUC from 0.927 to 1.000, indicating that polarization features provide information beyond intensity-derived descriptors.

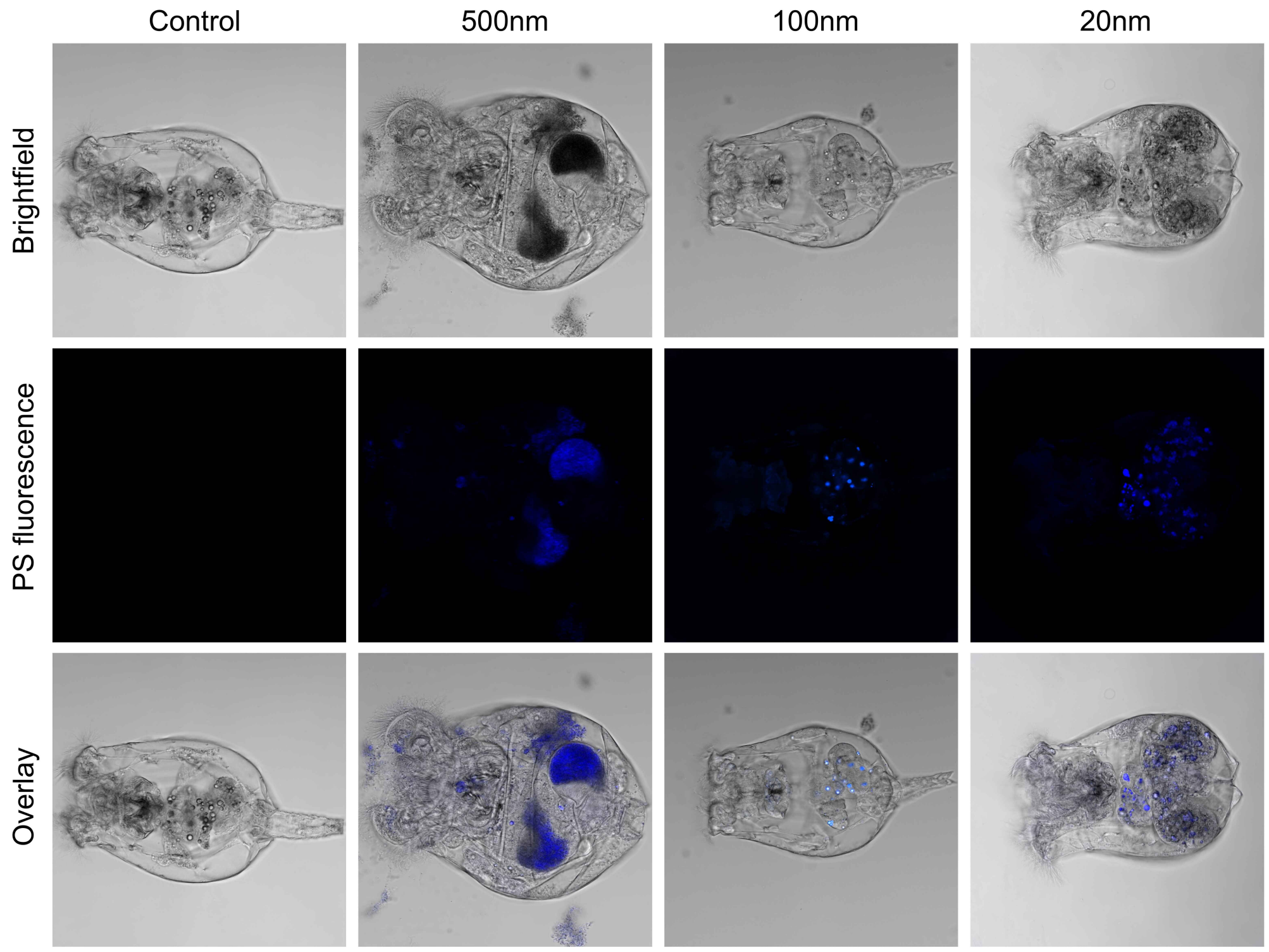

Figure S18: **Confocal validation of fluorescent PS nanoplastic burden in rotifers.** Representative brightfield, PS fluorescence, and overlay images of control and nanoplastic-exposed rotifers. Fluorescent PS signals are observed mainly within internal digestive regions.

Supplementary Tables

Table S1: Deep-learning ROI detector configuration and performance

Item	Description
Task	Rotifer ROI detection from raw holographic frames before numerical refocusing
Model architecture	Ultralytics YOLO11n lightweight object detector
Input image size	Original frame size: 1224 × 1024 pixels; network input size: 960 × 960 pixels
Input image type	Raw holographic intensity images
Annotation format	COCO-style bounding boxes
Number of annotated frames	600 frames
Number of annotated rotifer instances	22,739 total instances: 16,124 train, 4,378 validation, and 2,237 test instances
Training / validation / test split	420 / 120 / 60 frames, corresponding to 70% / 20% / 10% split by frame
Optimizer	Automatic optimizer selection in Ultralytics; AdamW selected
Initial learning rate	2.0×10^{-3}
Batch size	8
Training epochs	230 epochs
Evaluation metrics	Precision, recall, F1 score, mAP_{50}, and $mAP_{50:95}$
Detection performance	Precision = 0.961, recall = 0.948, F1 score = 0.954, mAP_{50} = 0.986, $mAP_{50:95}$ = 0.839
Inference hardware	NVIDIA GeForce RTX 4060 GPU

Table S2: **Physical properties and exposure metrics of PS nanoplastic beads at 5** mg L^{-1}

Diameter (nm)	Single bead volume (cm^3)	Individual mass (g)	Bead number in 1g	Bead number (mL^{-1})	Total surface area (m^2 L^{-1})
500	6.54×10^{-14}	8.18×10^{-14}	1.22×10^{13}	6.11×10^{7}	4.80×10^{-2}
100	5.23×10^{-16}	6.54×10^{-16}	1.53×10^{15}	7.65×10^{9}	2.40×10^{-1}
20	4.19×10^{-18}	5.23×10^{-18}	1.91×10^{17}	9.56×10^{11}	1.20

Table S3: **Kinematic thresholds used to define the six locomotor states**

State	Primary criterion	Operational definition
Freeze	Speed	$v < 0.2$ pixel frame^{-1} (< 10.9 μm s^{-1})
Slow swim	Speed	$0.2 \leq v < 1.0$ pixel frame^{-1} (10.9–54.3 μm s^{-1})
Normal swim	Speed	$1.0 \leq v < 2.5$ pixel frame^{-1} (54.3–135.8 μm s^{-1})
Rapid swim	Speed	$v \geq 2.5$ pixel frame^{-1} (≥ 135.8 μm s^{-1})
Turn	Turning angle	$\theta > 60°$
Meander	Turning angle	$30° < \theta \leq 60°$

Table S4: **Sample sizes and statistical units for quantitative analyses**

**Analysis**	**Condition**	*N*	*n*	**Statistical unit**
Behavioral-state analysis	Control	3	17	Single-organism trajectory
Behavioral-state analysis	500 nm	3	28	Single-organism trajectory
Behavioral-state analysis	100 nm	3	20	Single-organism trajectory
Behavioral-state analysis	20 nm	3	30	Single-organism trajectory
High-dimensional behavioral phenotyping	Control	3	162	Single-organism trajectory
High-dimensional behavioral phenotyping	500 nm	3	207	Single-organism trajectory
High-dimensional behavioral phenotyping	100 nm	3	190	Single-organism trajectory
High-dimensional behavioral phenotyping	20 nm	3	107	Single-organism trajectory
Polarization quantification	Control	3	15	Organism-level mean
Polarization quantification	500 nm	3	15	Organism-level mean
Polarization quantification	100 nm	3	15	Organism-level mean
Polarization quantification	20 nm	3	15	Organism-level mean

Note: N denotes independent biological replicates, and n denotes the number of statistical observations after quality filtering. For behavioral analyses, each observation represents one valid single-organism trajectory. Behavioral-state analysis includes temporal behavioral spectra, state occupancy, transition probability matrices, and behavioral-state entropy. High-dimensional behavioral phenotyping includes 27 behavioral descriptors, domain-level effect sizes, UMAP visualization, and the ecological warning index. For polarization quantification, each observation represents an organism-level mean calculated from three vesicle ROIs; individual frames, vesicles, and pixels were not treated as independent biological observations.

Table S5: **Technical specifications of the 4D tracking and high-resolution microimaging modes**

Parameter	High-throughput 4D tracking mode	High-resolution microimaging mode
Primary purpose	Volumetric tracking and reconstruction of 4D locomotor trajectories	High-resolution polarization mapping of structural anisotropy
Illumination	632.8 nm laser	Broadband halogen
Objective magnification	Lens-free	40×
Objective NA	N/A	0.65
Tube lens	N/A	1×
Camera model	PHX050S1-P/Q	PHX050S1-P/Q
Sensor subpixel size	3.45 μm	3.45 μm
Object-plane intensity pixel size	3.45 μm	0.086 μm
Effective polarimetric sampling	6.9 μm	0.173 μm
Field of view	8616 μm × 7192 μm	208 μm × 174 μm
Lateral resolution	7.81 μm	2.19 μm
Axial range	3000 μm	N/A
z-step / z-planes	100 μm	N/A
Axial localization precision	15.28 μm	N/A
Reconstruction latency	0.17 s per organism, median	N/A

Note: The optical configuration is modular. Objectives can be selected according to sample size, field-of-view requirement, and spatial-resolution demand. A 2×, NA 0.10 objective can be inserted for tracking, whereas a 100×, NA 1.25 objective can be used for higher-resolution microimaging.

Table S6: **Polarimetric calibration of AoP/DoLP accuracy and repeatability**

**Calibration item**	**Result**
Input linear-polarizer angles	0–170°, 10° interval
Complete four-channel image sets analyzed	180
Validation strategy	Leave-one-input-angle-out calibration
AoP bias	-0.001°
AoP MAE	0.50°
AoP RMSE	0.66°
AoP 95% absolute error	1.21°
Mean DoLP for linearly polarized input	1.000
DoLP MAE from the ideal value of 1	0.015
Within-angle AoP repeatability SD	0.025°
Within-angle DoLP repeatability SD	8.1×10^{-4}

Table S7: **Microplastic and nanoplastic particles' impairments on biological species**

Species	Concentration	Size	Polymer type	Exposure duration	Impairment	Reference
Rotifer *Brachionus plicatilis, B. calyciflorus*	5mg L^{-1}	5, 10, 20 and 30 µm	PS	4h	microplastic fragmentation	(*6*)
Rotifer *Brachionus koreanus*	0.1, 1, 10, and 20 mg L^{-1}	50 nm,500 nm, and 6 µm	PS	12days	oxidative stress, antioxidant enzymes, growth rate, fecundity, lifespan, reproduction time, body size	(*7*)
Rotifer *Brachionus plicatilis*	0.001, 0.01, 0.1, 1, 10 mg L^{-1}	100 nm	PS	6h	swimming behaviour, ingestion	(*8*)
Daphnia magna	50mg L^{-1}	20 and 200 nm	PS	21days	growth, molting, reproduction	(*9*)
Daphnia magna	16 µL L^{-1}	8.65 µm to 23.4 µm	PS	24h	swimming behaviour, body growth, survival rates	(*10*)
Mysid shrimp *Neomysis japonica*	10 mg L^{-1}	5 µm	PS	96h	swimming behavior, food uptake, lethal rate, growth inhibition	(*11*)
Zebrafish	0.1, 0.5, 1, 5, 10, 25, and 50 mg L^{-1}	80, 200, and 500 nm	PS	48-120h	developmental and neurobehavioral toxicities, oxidative stress, cell death and inflammation	(*12*)
freshwater cladoceran *Daphnia pulex*	2 mg L^{-1}	75 nm	PS	24h	growth, reproduction, stress defense	(*13*)
Drosophila melanogaster	0.01 to 100 ppm	1 µm, 20 nm	PS	8-13days	locomotion and daily activity	(*14*)
Copepod *Temora turbinata*	100 to 1000 beads/mL	20 µm	PS	24h	swimming behavior	(*15*)
Daphnia magna	0.05 and 0.5 µg mL^{-1}	50 nm	PS	21days	Molecular, biochemical and behavioral responses	(*16*)

Table S8: **Distribution of microplastics/nanoplastics in seawater**

Region	Location and depth	Coordinates	Concentration	Main Polymer(s)	Size (µm)	Reference
North Atlantic Ocean (mixed layer)	Subtropical gyre to European shelf, depth 10 m	8 − 55°N, 64°W − 4°E	18.1 ± 2.1 mg m^{-3}	PET, PS, PVC	< 1.0	(*17*)
North Atlantic Ocean (intermediate layer)	Same transect, depth 1000 m	8 − 55°N, 64°W − 4°E	10.9 ± 1.6 mg m^{-3}	PET, PS, PVC	< 1.0	(*17*)
North Atlantic Ocean (bottom layer)	Same transect, depth 30 m from seafloor	8 − 55°N, 64°W − 4°E	5.5 ± 0.6 mg m^{-3}	PET	< 1.0	(*17*)
North Atlantic Ocean (surface)	North–South transect, depth top 200 m	−60 − 60°N, −60 − 0°E	642 ± 916 µg m^{-3}	PE, PP, PS	32.0-651.0	(*18*)
Pacific Ocean (subtropical gyre)	Chinese sea area (the Bohai Sea), depth 25 m	39°N, 120°E	< 0.015 to 0.41 µg L^{-1}	PS	< 100	(*19*)
Dutch Wadden Sea	R/V Navicula cruise, depth 1 m and 3 m	53°N, 5°E	4.2 µg L^{-1}	PS	< 0.2	(*20*)
Mediterranean Sea (Adriatic, Venice Lagoon)	Venice–Lido port inlet, surface, depth 30 cm	45.431508°N, 12.406952°E	3.71×10^{7} particles mL^{-1}	not determined	mean 0.325	(*21*)
Mediterranean Sea (Adriatic, Venice Lagoon)	Grand Canal (under Rialto Bridge), surface, depth 30 cm	45.431508°N, 12.406952°E	7.8×10^{7} particles mL^{-1}	not determined	mean 0.387	(*21*)
Mediterranean Sea (Adriatic, Venice Lagoon)	Saint Marc basin, surface, depth 30 cm	45.431508°N, 12.406952°E	1.22×10^{8} particles mL^{-1}	not determined	mean 0.484	(*21*)
South China sea	Surface seawater, depth 20–50 cm)	9.76237°N, 111.9383°E	729 particles m^{-3}	PP, PE, PVC	100-500	(*22*)

References

1. J. W. Goodman, *Introduction to Fourier optics* (Roberts and Company publishers), 4 ed. (2005).

2. Y. Zhang, H. Wang, Y. Wu, M. Tamamitsu, A. Ozcan, Edge sparsity criterion for robust holographic autofocusing. *Optics Letters* **42** (19), 3824–3827 (2017).

3. S. Pertuz, D. Puig, M. A. Garcia, Analysis of focus measure operators for shape-from-focus. *Pattern Recognition* **46** (5), 1415–1432 (2013).

4. Z. Yan, G. Chen, W. Xu, C. Yang, Y. Lu, Study of an image autofocus method based on power threshold function wavelet reconstruction and a quality evaluation algorithm. *Applied Optics* **57** (33), 9714–9721 (2018).

5. Y. Gao, L. Cao, Iterative projection meets sparsity regularization: towards practical single-shot quantitative phase imaging with in-line holography. *Light: Advanced Manufacturing* **4** (1), 37–53 (2023).

6. J. Zhao, *et al.*, Microplastic fragmentation by rotifers in aquatic ecosystems contributes to global nanoplastic pollution. *Nature Nanotechnology* **19** (3), 406–414 (2024).

7. C.-B. Jeong, *et al.*, Microplastic size-dependent toxicity, oxidative stress induction, and p-JNK and p-p38 activation in the monogonont rotifer (Brachionus koreanus). *Environmental Science & Technology* **50** (16), 8849–8857 (2016).

8. C. Gambardella, *et al.*, Ecotoxicological effects of polystyrene microbeads in a battery of marine organisms belonging to different trophic levels. *Marine Environmental Research* **141**, 313–321 (2018).

9. O. Pikuda, *et al.*, Sub-lethal effects of nanoplastics upon chronic exposure to Daphnia magna. *Journal of Hazardous Materials Advances* **7**, 100136 (2022).

10. S. Magester, A. Barcelona, J. Colomer, T. Serra, Vertical distribution of microplastics in water bodies causes sublethal effects and changes in Daphnia magna swimming behaviour. *Ecotoxicology and Environmental Safety* **228**, 113001 (2021).

11. X. Wang, *et al.*, Polystyrene microplastics impaired the feeding and swimming behavior of mysid shrimp Neomysis japonica. *Marine Pollution Bulletin* **150**, 110660 (2020).

12. J. Chen, *et al.*, Polystyrene nanoplastics induced size-dependent developmental and neurobehavioral toxicities in embryonic and juvenile zebrafish. *Aquatic Toxicology* **267**, 106842 (2024).

13. Z. Liu, *et al.*, Polystyrene nanoplastic exposure induces immobilization, reproduction, and stress defense in the freshwater cladoceran Daphnia pulex. *Chemosphere* **215**, 74–81 (2019).

14. S. Matthews, *et al.*, Polystyrene micro-and nanoplastics affect locomotion and daily activity of Drosophila melanogaster. *Environmental Science: Nano* **8** (1), 110–121 (2021).

15. C. H. Suwaki, L. T. De-La-Cruz, R. M. Lopes, Impacts of microplastics on the swimming behavior of the copepod Temora Turbinata (Dana, 1849). *Fluids* **5** (3), 103 (2020).

16. B. De Felice, M. Sugni, L. Casati, M. Parolini, Molecular, biochemical and behavioral responses of Daphnia magna under long-term exposure to polystyrene nanoplastics. *Environment International* **164**, 107264 (2022).

17. S. Ten Hietbrink, D. Materić, R. Holzinger, S. Groeskamp, H. Niemann, Nanoplastic concentrations across the North Atlantic. *Nature* **643** (8071), 412–416 (2025).

18. K. Pabortsava, R. S. Lampitt, High concentrations of plastic hidden beneath the surface of the Atlantic Ocean. *Nature Communications* **11** (1), 4073 (2020).

19. Q. Li, *et al.*, Spatial distribution of polystyrene nanoplastics and small microplastics in the Bohai Sea, China. *Science of the Total Environment* **881**, 163222 (2023).

20. D. Materić, R. Holzinger, H. Niemann, Nanoplastics and ultrafine microplastic in the Dutch Wadden Sea–The hidden plastics debris? *Science of the Total Environment* **846**, 157371 (2022).

21. T. Cecchi, D. Poletto, A. C. Berbecaru, E. M. Cârstea, M. Râpă, Assessing Microplastics and Nanoparticles in the Surface Seawater of Venice Lagoon—Part I: Methodology of Research. *Materials* **17** (8), 1759 (2024).

22. Y. Cui, *et al.*, Microplastics in the surface waters of the South China sea and the western Pacific Ocean: different size classes reflecting various sources and transport. *Chemosphere* **299**, 134456 (2022).
